# Generalist Foundation Models Are Not Clinical Enough for Hospital Operations

**DOI:** 10.21203/rs.3.rs-9078142/v1

**Published:** 2026-03-19

**Authors:** Lavender Y. Jiang, Angelica Chen, Xu Han, Xujin Chris Liu, Radhika Dua, Kevin Eaton, Frederick Wolff, Robert Steele, Jeff Zhang, Anton Alyakin, Qingkai Pan, Yanbing Chen, Karl L. Sangwon, Daniel A. Alber, Jaden Stryker, Jin Vivian Lee, Yindalon Aphinyanaphongs, Kyunghyun Cho, Eric Karl Oermann

**Affiliations:** 1 Courant Institute School of Mathematics, Computing, and Data Science, New York University, 60 5th Ave, New York, 10001, NY, USA.; 2 Department of Neurosurgery, NYU Langone Health, 550 First Avenue, New York, 10016, NY, USA.; 3 Global AI Frontier Lab, New York University, 1 Metrotech Center, Fl. 22, Brooklyn, 11201, NY, USA.; 4 Electrical and Computer Engineering, Tandon School of Engineering, 6 MetroTech Center, Brooklyn, 11201, NY, USA.; 5 Grossman School of Medicine, New York University, 550 First Avenue, New York, 10016, NY, USA.; 6 Department of Medicine, NYU Langone Health, 550 First Avenue, New York, 10019, NY, USA.; 7 Department of Computer Science, ETH Zurich, Universitätstrasse 6, Zurich, 8092, Zurich, Switzerland.; 8 Department of Surgery, NYU Langone Health, 1 Park Avenue, New York, 10016, NY, USA.; 9 Division of Applied AI Technologies, NYU Langone Health, 227 East 30th Street, New York, 10016, NY, USA.; 10 Department of Population Health, NYU Langone Health, 450 First Avenue, New York, 10019, NY, USA.; 11 School of Medicine, Washington University of St. Louis, 660 S. Euclid Ave., St. Louis, 63110, MO, USA.; 12 School of Global Public Health, New York University, 708 Broadway, New York, 10003, NY, USA.; 13 Department of Radiology, NYU Langone Health, 450 First Avenue, New York City, 10019, NY, USA.

**Keywords:** pretraining, finetuning, Electronic Health Records, operational prediction, clinical prediction tasks, domain-specific models

## Abstract

Operational decisions governing patient flow, cost, and quality of care demand specialized predictive models, yet most clinical NLP efforts focus on medical knowledge benchmarks. We introduce Lang1, a family of language models (100M-7B parameters) pretrained on 80 billion clinical tokens from NYU Langone Health electronic health records blended with 627 billion internet tokens. We evaluate Lang1 on the REalistic Medical Evaluation (ReMedE), an evaluation suite derived from 668,331 Electronic Health Records (EHR) notes spanning five tasks: readmission, mortality prediction, length of stay, comorbidity coding, and insurance denial. In zero-shot settings, both general-purpose and biomedical models underperform on four of five tasks. After finetuning, Lang1-1B outperforms finetuned generalist models up to 70 × larger and zero-shot models up to 671× larger. Joint multi-task finetuning yields cross-task transfer, and Lang1-1B transfers effectively to unseen tasks and an external health system. These results demonstrate that effective healthcare AI requires in-domain pretraining, supervised finetuning, and evaluation beyond proxy benchmarks.

## 1 Main

Healthcare systems face high-stakes operational decisions daily: which patients are at imminent risk of decline, who can be safely discharged, how many beds will be available for new admissions. Physicians spend only 26% of their time in direct patient care, with much of the remainder devoted to documentation, insurance, and resource coordination [1–3]. Foundation models, with their broad text comprehension and applicability across specialized domains [4–6], are increasingly applied to healthcare. Yet despite strong performance on medical knowledge benchmarks, it remains unclear whether these generalist models can predict the operational outcomes that define day-to-day hospital care.

Deploying language models in clinical settings remains difficult. While models show promise on various clinical tasks [7–14], there is disagreement on whether smaller specialized models can outperform general-purpose models [15]. Many evaluations rely on proxy benchmarks that weakly reflect real-world clinical constraints like data scarcity and temporal shifts [9,16–19]. Data privacy concerns further limit clinical model pretraining to a small set of public corpora [20], even though large-scale EHR datasets are known to improve generalization [15, 21–23]. An earlier study showed that a 109M-parameter BERT model pretrained on clinical notes could predict operational outcomes such as readmission [23], but the field has since shifted with the rise of billion-parameter generalist models and instruction-tuned systems.

**Fig. 1:**
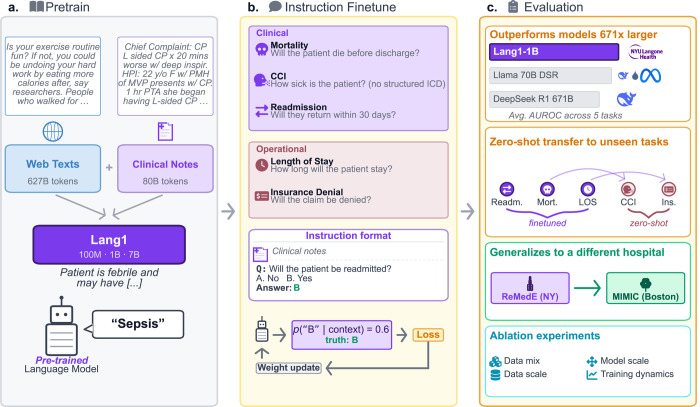
Overview of the Lang1 system. (a) We pretrain decoder language models on a mix of 627B tokens of web text and 80B tokens of clinical notes via next-token prediction. (b) Instruction finetuning in multiple-choice format activates clinical prediction capabilities and enables cross-task transfer. (c) Evaluation: Lang1-1B outperforms finetuned models up to 70× larger and zero-shot models up to 671× larger, transfers zero-shot to unseen tasks, generalizes across hospital systems, and yields design principles through ablation experiments.

Here we present Lang1, a family of decoder language models (100M, 1B, and 7B parameters) pretrained from scratch on a specialized corpus blending 80 billion tokens from the NYU Langone Health electronic health records (14.5 times more clinical data than the corpus used to train NYUTron [23]) with 627 billion tokens of internet text. To evaluate these models on clinically and operationally significant tasks, we developed the REalistic Medical Evaluation (ReMedE), an evaluation suite derived from 668,331 EHR notes spanning five clinically and operationally significant tasks: 30-day readmission prediction, in-hospital mortality prediction, length of stay, comorbidity coding, and insurance claims denial (Figure 1). Unlike benchmarks that focus on diagnostic reasoning [7, 13, 16], ReMedE emphasizes operational tasks tied to resource planning, cost control, and continuity of care. Each task is evaluated on data from the future relative to all training data, approximating deployment conditions. After task-specific finetuning, Lang1-1B outperforms finetuned models up to 70x larger (LoRA-finetuned DeepSeek R1 Distill Llama 70B) on ReMedE. In the commercially relevant zero-shot setting, where hospitals evaluate models via API without capacity for finetuning, it also surpasses generalist models up to 671 × larger, including DeepSeek R1. Instruction-finetuned on one or more tasks, Lang1 transfers zero-shot to related tasks and to a different hospital system, surpassing both generalist and open biomedical models of similar scales. Our analysis of training dynamics reveals that clinical prediction does not emerge from pretraining alone but requires task-specific supervision, which in-domain pretraining makes more data-efficient. Overall, our results suggest that health systems with the capacity for in-house model development can gain clear advantages from smaller specialized models, providing a practical and data-efficient pathway to robust operational prediction with minimal task-specific supervision.

Unlike encoder-based clinical models such as NYUTron [23] that require separate classification heads per task, Lang1 uses a decoder architecture that supports instruction finetuning across tasks in a unified multiple-choice format. Our approach consists of three stages (Figure 1): pretraining on a mix of web text and clinical notes (Fig. 1a), instruction finetuning in multiple-choice format (Fig. 1b), and evaluation against generalist models with ablation experiments (Fig. 1c). See Methods for full details.

We evaluate Lang1 using ReMedE, an internal evaluation suite of real-world, high-impact clinical tasks beyond diagnosis. Unlike recent benchmarks that focus on multi-turn diagnostic dialogue [7, 13, 16], which captures an important but narrow part of clinical decision making, ReMedE is based on 668,331 EHR notes and emphasizes operational tasks that better represent the day-to-day challenges of healthcare delivery. To assess model robustness to temporal distribution shifts, each task is evaluated across three non-overlapping test splits drawn from distinct time periods (Extended Data A). We plan to release ReMedE as a secure evaluation service, allowing trusted researchers to submit models and receive standardized evaluation results without direct access to patient data.

## 2 Results

### 2.1 Overall performance on ReMedE

#### Finetuned Lang1 outperforms larger zero-shot and finetuned models.

**Fig. 2:**
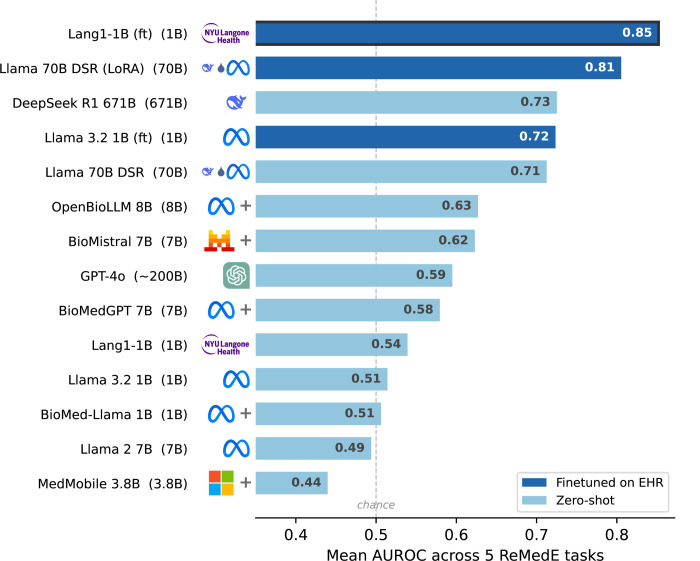
Finetuned small specialists outperform strong generalists on ReMedE. Mean Area Under the Receiver Operating Characteristic curve (AUROC) across five clinical tasks for 14 models, ranked by performance. Finetuned Lang1-1B (1B parameters) achieves the highest average performance (0.85), outperforming all zero-shot models up to 671B parameters. Per-task breakdowns and the full heatmap are in Extended Data B.

After finetuning, Lang1-1B achieves a mean AUROC of 85.2% (range 76.7%−95.9%) across the five ReMedE tasks, surpassing finetuned Llama 3.2 IB (72.4%, range 64.4%−78.8%), LoRA-finetuned DeepSeek R1 Distill Llama 70B (80.5%, range 70.4%−91.5%), and zero-shot DeepSeek RI 671B (72.5%, range 60.4%−94.2%) across all five tasks (Figure 2). Across all five tasks, Lang1-1B achieves higher AUROC than every baseline (ranking and per-task breakdowns in Extended Data B). Scaling to Lang1-7B yields only marginal gains (Supplementary L), suggesting model size is not the bottleneck at this data scale.

#### Large generalist models underperform on real-world clinical predictive tasks.

For hospitals evaluating commercial APIs without capacity for finetuning, the zero-shot gap is even wider. We evaluated 14 models (spanning large generalist foundation models, MedQA leaderboard models, and biomedical specialists) under zero-shot inference and find that they underperform on ReMedE tasks (Figure 2). This includes GPT-4o, which achieves a mean AUROC of only 59.5% (range 49.4%−69.8%) using sampling-based probability approximation (Supplementary S). Even the best zero-shot model (DeepSeek RI 671B) scores only 60.4%−71.1% AUROC on four of five tasks; mortality is the exception at 94.2%, but still 1.7 percentage points below finetuned Lang1-1B.

### 2.2 Clinical prediction does not emerge from pretraining

#### Unlike reading comprehension, clinical classification does not emerge from pretraining.

**Fig. 3:**
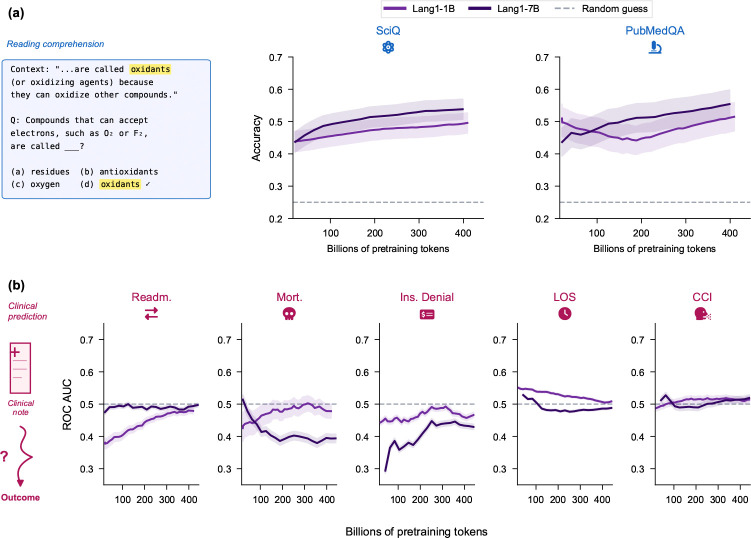
Clinical classification does not emerge from pretraining, unlike reading comprehension. (a) Reading comprehension accuracy increases during pretraining. (b) Zero-shot clinical AUROC remains near chance throughout pretraining. Error bands depict 95% confidence intervals.

We tracked zero-shot performance of Lang1 (1B and 7B) throughout pretraining as a function of tokens seen. On comprehension tasks (Methods 4.2.4), accuracy increased with additional pretraining data (Figure 3a), consistent with the expectation that language models improve on text-based reasoning with more data. In contrast, zero-shot AUROC on ReMedE clinical classification tasks remained close to or below random chance across the entire pretraining trajectory (Figure 3b). The mapping from clinical notes to operational outcomes does not emerge from next token prediction on unlabeled text alone but needs to be learned through task-specific finetuning. This is not simply a matter of the base model failing to follow instructions: applying general-purpose instruction finetuning on OASST2 before evaluation does not improve clinical classification (Supplementary J).

#### Finetuning is more token-efficient, but pretraining makes finetuning more data-efficient.

**Fig. 4:**
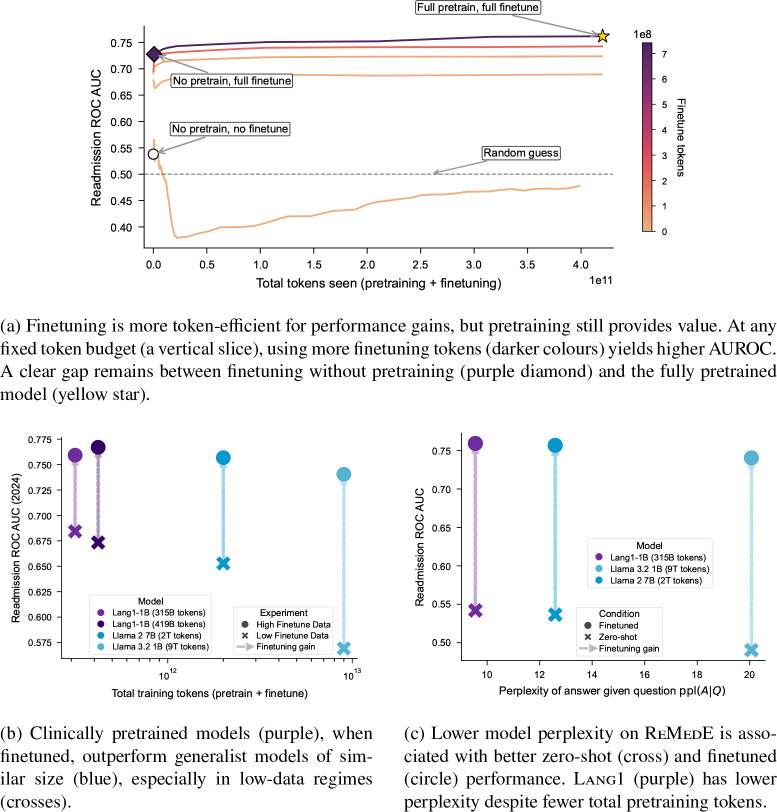
In-domain pretraining makes finetuning more efficient. (a) At any fixed total token budget, more finetuning tokens yield higher AUROC, but pretraining still makes finetuning more data efficient. (b) In-domain pretraining enables sample-efficient finetuning. (c) Lower perplexity on clinical tasks is associated with better performance. Error bars depict 95% confidence intervals.

Figure 4a examines the pretraining and finetuning trajectory of Lang1-1B for readmission under a fixed total token budget. During pretraining, checkpoints were saved after each one million training tokens. Each pretrain checkpoint is finetuned using 100–362,259 discharge notes with readmission label (2.0M-742.0M tokens). Within each budget slice, increasing the proportion of finetuning tokens consistently improves performance. Yet pretraining still provides value: even with maximal finetuning data, models initialized from pretraining outperform randomly initialized ones by 4.71% AUROC. The same pattern holds across all five tasks (Supplementary I). When finetuning data are scarce (Figure 4b), Lang1-IB outperforms generalist models of comparable scale pretrained on more nonclinical tokens, demonstrating that in-domain pretraining reduces the number of labelled examples required to 6 reach a given performance level. Lang1-1B also achieves lower perplexity on clinical task pairs and stronger downstream performance than Llama-2-7B and Llama-3.2-1B, despite being trained on fewer total tokens (Figure 4c). This correlation between perplexity and finetuned performance, especially in the low-data regime, suggests that task-specific perplexity may serve as a practical heuristic for model selection before committing to finetuning. Adaptation on published biomedical text does not substitute for pretraining on clinical notes. Lang1-1B outperforms Bio-Medical-Llama-3.2-1B, a continually pretrained variant of Llama-3.2-1B, at every sample size from 100 to 362,259, with the largest gains (17.9 percentage points) at 100 examples (Supplementary K). Additional pretraining ablations show that larger models trained on more recent clinical data further improve performance (Supplementary L).

### 2.3 Transfer across tasks and health systems

#### A single model handles all five tasks and transfers across them.

**Fig. 5:**
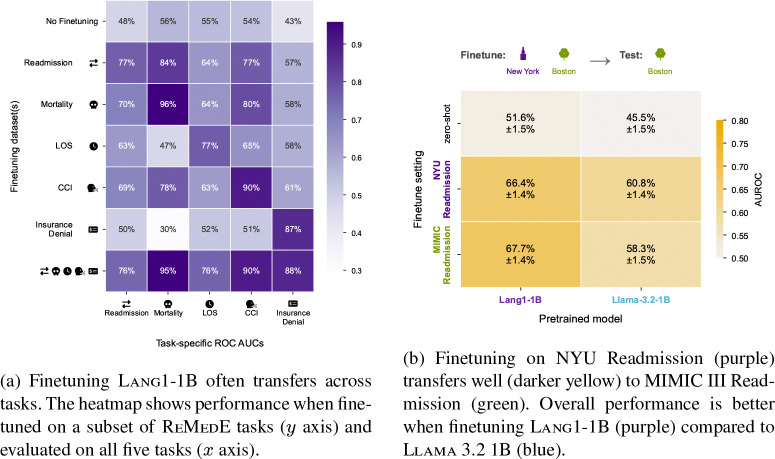
Lang1 transfers to unseen tasks and a different health system. (a) Cross-task transfer heatmap for Lang1-1B on ReMedE. (b) External validation on MIMIC III readmission.

The heatmaps in Figure 5a show Lang1-1B finetuned on one or all ReMedE tasks (rows) and evaluated on all five tasks (columns). Lang1-1B achieves strong single-task (diagonal) performance, and a single model jointly finetuned on all five tasks (last row) comes within 1 pp of single-task performance on every task, reducing the need to train and maintain separate models for each operational task. Both single-task and jointly finetuned Lang1 models are well calibrated across all five tasks, with calibration curves closely tracking the diagonal and low expected calibration error (Extended Data C). Many tasks also transfer individually: finetuning on readmission alone boosts performance on the other four tasks. However, this transfer can be asymmetric: mortality helps LOS, but LOS does not help mortality, which can be explained by the conditional probability structure of these outcomes (Supplementary M). Transfer patterns are model-specific, and Lang1-1B transfers best: compared to Llama-3.2-1B (Extended Data D) and Bio-Medical-Llama-3.2-1B (Supplementary K), Lang1-1B has the highest single-task, off-diagonal, and joint performance. Joint finetuning drops only 0.4 percentage points (pp) from single-task performance for Lang1-1B, compared to 3.4 pp for Bio-Medical-Llama and 10.4 pp for Llama-3.2-1B, suggesting that clinical note pretraining confers greater multi-task robustness than biomedical literature or general-domain pretraining.

#### Lang1 transfers to an external health system.

Figure 5b shows Lang1-1B and Llama-3.2-1B finetuned on different readmission data (MIMIC III, derived from Beth Israel Deaconess Medical Center in Boston, or NYU in New York) and tested on MIMIC. Finetuning Lang1-1B yields better performance on both datasets. For Lang1-1B, finetuning on MIMIC is slightly better than NYU by 1.2% AUROC. For Llama-3.2-1B, finetuning on NYU is surprisingly better than finetuning on MIMIC by 2.5% AUROC, likely because NYU has more labeled pairs, suggesting that nonclinical models may benefit more from larger, slightly out-of-distribution datasets. Extended analysis on MIMIC mortality and LOS shows consistent findings: Lang1-1B outperforms Llama-3.2-1B and NYU-finetuned models transfer well to MIMIC (Extended Data E).

## 3 Discussion

NYUTron [23] demonstrated that LLMs can improve hospital operations, but required a separate model and labeled dataset per task, with no cross-task transfer. Since then, frontier models such as GPT-4o and open-weight models such as Llama have expanded the options available to hospitals, creating a spectrum of approaches that range in effort and cost from zero-shot prompting of large models, to parameter-efficient finetuning, to full finetuning, to continual pretraining, to pretraining from scratch. We systematically evaluate the approaches realistic for hospital deployment, from zero-shot through LoRA finetuning of 70B-parameter models to full pretraining at 100M, 1B, and 7B parameters, quantifying the marginal gain from each additional investment. Using a single jointly finetuned Lang1 model that handles all five tasks and transfers across tasks and health systems, we find that clinical prediction does not emerge from pretraining alone, that domain-specific pretraining improves data efficiency and cross-task transfer, and that small specialized models can match much larger generalists. Together, these results suggest that investing in domain-specific pretraining, even at modest scale, yields a single model that outperforms much costlier alternatives.

### Operational tasks require direct evaluation, not proxy benchmarks.

Much of the current excitement in medical AI centers on diagnostic reasoning [16, 24–27]. These are valuable directions, but they do not fully capture the day-to-day challenges physicians face: physicians spend only 26% of their time in direct patient care, with much of the remainder devoted to documentation, insurance, and scheduling [1]. Operational outcomes such as readmission, insurance denial, and length of stay directly shape costs, capacity, and continuity of care, yet are poorly represented in web-scale datasets. Several MedQA leaderboard models underperform on ReMedE, showing that proxy benchmark success does not necessarily establish clinical utility and that models must be evaluated directly on real-world, task-specific outcomes [9,19]. All ReMedE results are reported on a 2024 temporal test set drawn from a period after all pretraining data, approximating the distribution shift encountered in deployment.

### Clinical prediction requires finetuning and does not emerge from pretraining alone.

While reading comprehension capabilities emerge directly from large-scale pretraining, our findings provide evidence that high-stakes objective predictions represent a different class of problem. Strong performance on ReMedE tasks requires explicit finetuning and does not emerge from pretraining alone, even with domain-specific data. Chatbot finetuning aligns emergent generalist skills to *subjective*, preference-based goals [28]; in contrast, ReMedE tasks require finetuning to build a new, non-emergent predictive skill against an *objective*, ground-truth target. The one exception is mortality, where zero-shot models reach 94.2% AUROC. We hypothesize this reflects how mortality signals closely mirror published medical literature and case reports present in web-scale pretraining corpora.

### Finetuning enables transfer across tasks and health systems.

Healthcare tasks often suffer from limited labels due to the expertise required for annotation, and in some cases large labeled datasets are practically impossible to obtain. Instruction finetuning on one outcome (e.g., readmission) improves performance on others (e.g., mortality, length of stay), and models finetuned on NYU data transfer to MIMIC III with minimal degradation (Extended Data E), reducing dependence on costly annotation pipelines. Notably, clinical note pretraining also confers multi-task robustness: Lang1-1B’s jointly finetuned model loses only 0.4 pp relative to single-task models, compared to 3.4 pp for Bio-Medical-Llama and 10.4 pp for Llama-3.2-1B, suggesting that clinical note pretraining better enables multi-task learning.

### Lang1 matches NYUTron while enabling multi-task and cross-site transfer.

NYUTron [23] established the clinical utility of language-model-based operational prediction on the same tasks and patient population, including prospective deployment and comparison against established structured baselines. NYUTron outperformed these baselines by 5–15% AUROC across tasks. Lang1 achieves a slightly higher average AUROC than NYUTron across all five tasks (Extended Data K), while additionally supporting multi-task learning and cross-hospital transfer from a single model. For fair comparison with NYUTron, all finetuning inputs are right-truncated to 512 tokens; zero-shot evaluation at full sequence length shows no statistically significant difference in AUROC and preserves model rankings (Extended Data H).

### Specialized models are cost-effective and keep data in-house.

Training Lang1-1B on 314.5B tokens required roughly 30 days on 64 H100s, costing about $180,000 at cloud pricing, which is orders of magnitude below frontier model budgets [4, 5, 29] and comparable to a routine IT infrastructure upgrade for a large health system. Continual pretraining from an existing open-source checkpoint can further reduce entry costs (Figure F8). However, this efficiency comes with a tradeoff since continual pretraining plateaus earlier and the benefit of the general-domain initialization is itself task-dependent (Extended Data F). Institutions must therefore weigh faster, cheaper convergence against the long-run gains of a full training run. Beyond cost, in-house models allow hospitals to safeguard patient data, adapt to documentation practices and patient populations, and avoid ongoing dependence on external APIs. Routing identifiable clinical notes through external APIs raises privacy risks: even HIPAA-compliant de-identification may not prevent re-identification by the very LLMs processing the data [30]. In-house models like Lang1 sidestep this problem as data never leaves the institution’s secure infrastructure and model internals remain auditable. This supports recent arguments [31] that small, specialized models can be more reliable, economical, and aligned with domain-specific needs, reframing clinical AI from “renting intelligence” to “building institutional assets.”

### Models are well calibrated with subgroup variations consistent with prior work.

Both single-task and jointly finetuned Lang1 models are well calibrated across all five tasks (Extended Data C), meaning predicted probabilities reliably reflect true outcome rates. We performed stratified evaluation of readmission prediction: the task most likely to trigger differential clinical interventions. We found that Lang1 performs above chance across all subgroups (Extended Data G), with moderate variation consistent with the patterns reported by NYUTron [23] on the same patient population. For insurance denial prediction, the intended use is proactive (identifying documentation gaps before claim submission to reduce denials) but dual-use risks exist (e.g., patient selection) and institutional safeguards are essential. Lang1 predictions should be integrated as decision support with clinician oversight and prospective monitoring.

### Smaller, domain-specific models offer a practical path forward for clinical AI.

Our findings challenge the assumption that ever-larger internet-trained models will generalize to all domains. A single finetuned Lang1-1B outperforms frontier models orders of magnitude larger, handles all five tasks with minimal degradation, transfers across tasks without additional labels, and generalizes to an external health system. Hospitals today face a concrete choice among commercial APIs, open-weight finetuning, or domain-specific pretraining. Our results show that the last path wins: better performance, a single model across tasks, and data that never leaves the institution. Where NYUTron [23] opened the door to language-model-based operational prediction, Lang1 shows that a hospital can train its own small model and surpass what much larger general-purpose models can offer. We believe effective healthcare AI need not depend on ever-larger models, and that hospitals can build their own institutional AI assets that are accurate, affordable, and under their full control.

## 4 Methods

### 4.1 Data collection and preprocessing

Data are extracted via SQL scripts from the NYU Langone Health EHR, prototyped in an interactive web-based editor (Cloudera Hue), and exported as CSVs to an on-premises high-performance computing cluster. Raw CSV notes (including pathology, radiology, and general hospital notes) are loaded with standard ASCII encoding using Python Dask [32] for distributed processing. We concatenate narrative fields, standardize punctuation, spacing and formatting via regular expression substitutions, remove non-ASCII and malformed characters, remove errant whitespace and newlines, and filter out short notes (less than 10 words or with placeholder values such as <NA>).

### 4.2 Datasets

#### 4.2.1 Pretraining dataset

##### Web texts.

We use SlimPajama (627B tokens) [33], a large, extensively deduplicated, multi-corpora, open-source dataset for training LLMs. Its sources include CommonCrawl [34], C4 [35], GitHub, Books [36, 37], arXiv, Wikipedia, and StackExchange.

##### NYU Notes.

This dataset consists of unlabeled inpatient hospital notes signed by medical professionals from the NYU Langone Health EHR^1^ for patient encounters from January 2011 to May 2020. NYU Notes contains 387,144 patients, 7,247,694 notes, and 4,112,249,482 words. NYU Notes was used to train and evaluate NYUTron [23].

##### NYU Notes+.

This dataset builds on NYU Notes by including a wider range of note types and covering a longer time span, resulting in a total word count 14.5 times greater than NYU Notes, the dataset used to train NYUTron [23]. NYU Notes+ contains unlabeled hospital, pathology, and radiology notes from the NYU Langone Health EHR from 2003 to 2023. It comprises 11,689,342 patients, 180,487,092 notes, and 59,917,646,788 words.

#### 4.2.2 Finetuning datasets and ReMedE test set

We derive five task-specific labelled datasets by combining NYUTron [23] finetuning datasets with the addition of a 2024 temporal test set to approximate deployment robustness. The 2024 temporal test set is used for ReMedE. See Extended Data A for a visualization of the data split timeline and Supplementary N for detailed dataset statistics. Supplementary O shows that a small percentage of patient overlap does not overestimate model performance on readmission. For both zero-shot evaluation and finetuning (Section 4.4), the datasets are converted to multiple choice format (Supplementary P).

##### NYU+ Readmission.

Readmission occurs when a patient returns to the hospital shortly after discharge. Predicting readmissions is critical for identifying patients who need longer stays or post-discharge support, and it serves as a key hospital quality metric. This dataset contains discharge notes with 30-day all-cause readmission labels. The notes comprise a subset of NYU+ Notes whose encounters end between January 2013 and November 2021, with additional discharge notes from 2024 for the temporal test. Rehabilitation, dialysis, and palliative care notes are excluded to focus on modelling acute readmission. A positive label is assigned if the patient is readmitted within 30 days of discharge, and a negative label otherwise. We split the dataset into five sets: train, validation, and test (8:1:1 ratio, 2013 to May 2021), 2021 temporal test (June to December 2021), and 2024 temporal test. The positive class ratio ranges from 10.81% to 11.29%. The dataset contains 421,429 patients, 604,326 notes, and 607,877,177 words.

##### NYU+ In-Hospital Mortality.

In-hospital mortality prediction identifies patients at highest risk of death during admission, enabling timely palliative care consultations and goals-of-care discussions. This dataset contains history and physical (H&P) notes with in-hospital mortality labels. A positive label is assigned if the discharge disposition is “Expired”. The dataset contains 395,991 patients, 566,748 notes, and 608,603,182 words. The positive class ratio ranges from 1.78% to 1.93%.

##### NYU+ Length of Stay (LOS).

LOS is the number of days a patient remains hospitalized. Predicting LOS is essential for bed management, staffing allocation, and discharge planning. This dataset contains H&P notes with binned LOS labels assigned by quantile: 0–2 days (<25th percentile), 3 days (25th-50th), 4–5 days (50th-75th), and >5 days (>75th). The dataset contains 395,991 patients, 566,748 notes, and 608,603,182 words.

##### NYU+ Insurance Denial.

Insurance denials occur when payers reject claims for hospital services. Predicting denials allows hospitals to proactively address documentation gaps, reducing administrative burden and preventing unexpected out-of-pocket costs for patients. This dataset contains H&P notes with insurance denial labels for encounters ending between May 2021 and April 2022, with additional H&P notes from January 2024 for the temporal test. The positive class ratio ranges from 12.01% to 13.90%. The dataset contains 87,974 patients, 97,837 notes, and 89,147,715 words.

##### NYU+ Charlson Comorbidity Index (CCI).

CCI is a standard score used to quantify a patient’s chronic illness burden based on medical history [38]. This dataset provides H&P notes paired with binned CCI scores computed from International Classification of Diseases (ICD) codes [39]. The CCI is discretized into five classes: 0, 1–2, 3–4, 5–7, and >7. The dataset contains 306,741 patients, 443,915 notes, and 524,739,038 words.

#### 4.2.3 External validation datasets

We create external validation datasets from MIMIC III [40], sourced from Beth Israel Deaconess Medical Center in Boston.

##### MIMIC III Readmission.

The labelled dataset has 6% positive labels, with 52,725 examples and a 70%/15%/15% train/validation/test split. Dataset construction details are in [41].

##### MIMIC III Mortality.

The labelled dataset has 10.55% positive labels, with 5,658 examples and an 80%/10%/10% split. We identify admission notes by filtering note descriptions, select one note per hospital stay using a prioritization heuristic, and remove notes written > 120 hours after admission.

##### MIMIC III LOS.

The labelled dataset uses the same 5,658 admission notes as mortality, with a mean LOS of 7.96 days. Continuous LOS values are discretized using the NYU+ LOS scheme.

#### 4.2.4 Comprehension datasets

We evaluate the performance of Lang1 checkpoints on comprehension datasets to analyse the emergence of nonclinical abilities.

**SciQ** [42]. Contains 13.7K multiple choice science exam questions with contexts.

**PubMedQA** [43]. Contains 1K expert-annotated biomedical question-answering examples from PubMed abstracts.

### 4.3 Pretraining Lang1

We pretrain a family of Llama-style decoders (Lang1-100M, Lang1-1B, Lang1-7B) on a mixture of web texts and NYU Notes+ (Section 4.2.1) using next token prediction (Figure 1a). Detailed demographic statistics are in Supplementary Q. Unless otherwise noted, Lang1 models are trained with equal sampling from both clinical and general sources, which is supported by our pretraining ablations (Supplementary L). For tokenization, we use the Llama-2-7B tokenizer (SentencePiece, 32K vocabulary). The 100M-parameter model follows the Smol-Llama-101M architecture with a 1,024 context length; the 1B model follows TinyLlama-1.1B with a 2,048 context length; and the 7B model follows Llama-2-7B with a 4,096 context length.

We pretrain on 8 to 64 NVIDIA 80GB H100 GPUs with NVLink, using the LitGPT [44] library and Fully Sharded Data Parallel [45]. We run manual hyperparameter search trials based on speed, performance, and training stability. For all models we use AdamW with linear warmup (2,000 steps), *β*_1_ = 0.9, *β*_2_ = 0.95, *ϵ* = 10^−8^, and cosine decay to a minimum learning rate of 4 × 10^−5^. We use a seed of 3407, weight decay of 0.1, and gradient clipping of 1. We shard gradient and optimizer for models up to 1B, and apply full sharding for the 7B model. The effective batch size is 4,096 for the 100M model and 1,024 for the 1B and 7B models.

We implement a monitoring pipeline that automatically triggers few-shot evaluations and generations at fixed pretraining intervals. Alerts are configured to report loss spikes. Upon detection of anomalies, we revert to the most recent stable checkpoint. Validation loss is computed periodically on a held-out 0.1% split and used for checkpoint selection.

#### 4.3.1 Pretrained models

We pretrain the variants listed in Table 1. Ablations (Supplementary L) show that larger models trained on more clinical data perform better, and that mixing in web texts does not substantially hurt downstream performance. When we refer to Lang1 without specifying data sources, we mean the variant trained with NYU Notes+ and web texts.

### 4.4 Finetuning

We finetune Lang1 models (and their trajectory of checkpoints) and other pretrained models (Table 2) on ReMedE tasks using multiple choice format (Figure 1b). The labelled clinical notes are converted to multiple choice format (Supplementary P), and we train the model to predict the correct option. For fair comparison with NYUTron, we right-truncate all clinical notes to a maximum of 512 tokens. Zero-shot evaluation at native context lengths confirms this truncation does not affect model rankings or conclusions (Extended Data H). All finetuning jobs use one node of 8 NVIDIA 80GB H100 GPUs.

Before each full finetuning run, we conduct 5 hyperparameter search trials up to 100 steps using Hydra and Optuna. We search learning rate in log scale over [10^−6^, 10^−3^] [46]. We use AdamW with *β*_1_ = 0.9, *β*_2_ = 0.999, *ϵ* = 10^−5^, weight decay of 0.02, no gradient clipping, and cosine annealing with no warmup for a maximum of 5,120 steps. The best trial is selected based on validation AUROC and loss.

For full finetuning, we use the best learning rate and train for a maximum of 5,120 steps with early stopping based on Micro-AUROC (patience of 300 steps). Probabilities for AUROC are obtained by normalizing the logits of the multiple choice options. We train all parameters except for DeepSeek-R1-Distill-Llama-70B, which is finetuned using low-rank adaptation [47] to meet memory constraints (Supplementary R). For multitask finetuning, we mix examples from each task evenly within each training batch, scaling total steps by the number of tasks.

### 4.5 Evaluation

#### Pretraining evaluation.

We monitor token-level cross-entropy loss and perplexity for both training and validation.

#### Zero-shot and few-shot evaluation.

ReMedE is built on the LM Eval Harness [48]. We implement the tasks as multiple choice questions with AUROC as the metric and a child class of LocalCompletionsAPI to connect on-premises models. For models whose logits are not accessible (e.g., on-premises GPT-4o), we implement a custom sampling function to approximate probabilities (Supplementary S) by counting choices from 10 generations at temperature 1.

#### Finetuning evaluation.

We collect the logits of the multiple choice options, normalize them as probabilities, and calculate AUROC using scikit-learn (consistent with ReMedE’s backend). For multiclass classification, we use One-Versus-Rest (OVR) AUROC.

#### Uncertainty.

We calculate 95% confidence intervals (CI = ± 1.96 x standard deviation) by resampling each test set 1,000 times using the quantile bootstrap method from SciPy. Bootstrap CIs assume independence of test instances; Extended Data O confirms that the small fraction of recurring patients does not materially affect conclusions (< 1 pp change). Non-overlapping 95% CIs between two models conservatively imply *p* < 0.05; all key comparisons reported in this paper (e.g., Lang1-1B vs. Llama 3.2 1B, Lang1-1B vs. LoRA-finetuned Llama 70B) meet this criterion.

#### Covariates.

Main finetuning and zero-shot analyses do not adjust for demographic covariates; results stratified by age, sex, race, ethnicity, borough, and pediatric status are presented in Extended Data G for transparency on differential performance.

#### Temporal shift.

To better approximate deployment conditions under temporal distribution shift, all AUROCs are reported on test data from 2024, drawn from a period after the pretraining data, unless otherwise noted. See Extended Data A for a visualization.

#### Generalist models.

We compared against generalist frontier models, including DeepSeek RI (served via vLLM [49]), DeepSeek RI Distilled Llama 70B (vLLM), and on-premises GPT-4o (Azure-hosted). Additional models (Llama 3.3 70B Chat) were evaluated in the context length comparison (Extended Data H). We also evaluated MedQA leaderboard models including Llama 3.2 IB, Llama 2 7B, and MedMobile.

## Supplementary Material

Supplement 1

## Figures and Tables

**Table 1:** Pretrained model specifications.

Model Name	Model Size	Pretrain Data
Lang1-100M-NYUNotes	100M	NYU Notes
Lang1-100M-NYUNotes+	100M	NYU Notes+
Lang1-100M-NYUNotes+,WebTexts	100M	NYU Notes+, Web Texts
Lang1-1B-NYUNotes	1B	NYU Notes
Lang1-1B-NYUNotes+	1B	NYU Notes+
Lang1-1B-NYUNotes+,WebTexts	1B	NYU Notes+, Web Texts
Lang1-7B-NYUNotes+,WebTexts	7B	NYU Notes+, Web Texts

**Table 2:** Additional model specifications.

Model Name	Model Size	Pretrain Data
Llama-3.2-1B	1B	Unnamed public mix (9T tokens)
Llama-2-7B	7B	Unnamed public mix (2T tokens)
DeepSeek-R1-Distill-Llama-70B	70B	Public mix (2T tokens) + reasoning data

## Data Availability

The clinical data used for the pretraining, finetuning, validation, and test sets were collected from the NYU Langone Health System EHR maintained by the NYULH Datacore team. Text data was stripped of rich text features and directly included in the dataset “as-is”, and was augmented with structured features where noted. It consists of the production medical records of NYU Langone and cannot be made publicly available. For the external validation task, the datasets were obtained from MIMIC III, and are publicly available from their website.

## Footnotes

1 This study is approved by the Institutional Review Board (IRB) at NYU Langone Health. The methods are carried out in accordance with the IRB’s relevant guidelines and regulations.

## Code Availability.

This work uses several open-source libraries including PyTorch, LitGPT, Transformers library, LM Eval Harness, and Hydra. Our experimental framework involves the utilization of these libraries and in some cases modification of them. We will release code to replicate the pretraining, finetuning, and testing of the models described in this paper at the time of publication. We include detailed methods and implementation steps in the Methods and Supplementary Information to allow for independent replication.
