# Supplementary material for: Generalist Foundation Models Are Not Clinical Enough for Hospital Operations
Source: Res Sq. 2026 Mar 19:rs.3.rs-9078142. Preprint. [Version 1] doi: 10.21203/rs.3.rs-9078142/v1 (PMC13015597; doi:10.21203/rs.3.rs-9078142/v1)
Supplement: Supplement 1 [file NIHPPRS9078142V1-supplement-1.pdf]

Extended Data A Data Timeline

LANG1's pretraining data covers a wider time window than NYUTRON [23]. Figure A1a illustrates the data timeline. NYUTRON's pretraining data spans 2013 to May 2021. LANG1's pretraining data covers 2003 to 2023. Overall, LANG1's pretraining corpus is more than 10 times the size of NYUTRON.

LANG1 adds additional temporal test sets. For both NYUTRON and LANG1 finetuning, we use temporal test sets to approximate deployment conditions. For NYUTRON, the temporal test set is from June to December 2021. LANG1 uses both the June–December 2021 temporal test set and an additional 2024 temporal test set. All main-text performance is reported on the 2024 temporal test set unless otherwise specified.

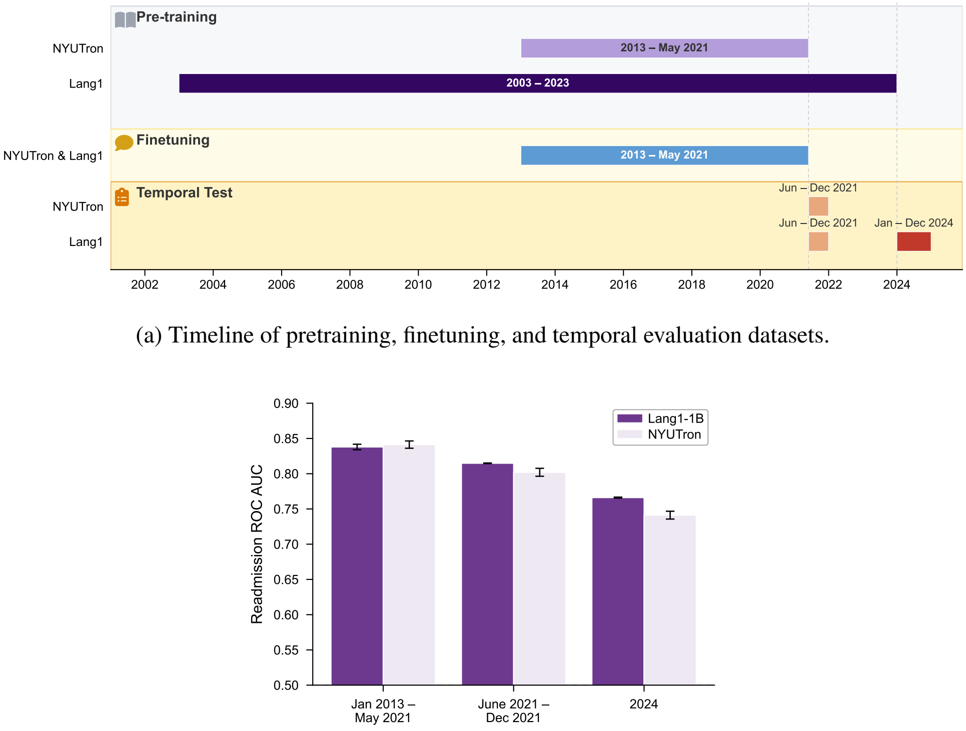

(a) Timeline of pretraining, finetuning, and temporal evaluation datasets.

(b) Models perform worse on temporal test sets.

Fig. A1: **Data timeline and temporal degradation.** (a) LANG1 covers a wider pretraining window (2003–2023 vs. 2013–2021) and evaluates on both 2021 and 2024 temporal test sets. (b) Both LANG1-1B and NYUTRON degrade on more distant temporal test sets.

Temporal test is important and difficult. Both LANG1-1B (purple) and NYUTRON (pink) perform worse as test data are sampled from further in the future (Figure A1b), illustrating the importance of evaluating with temporal test sets.

Extended Data B Full Model Comparison on ReMedE

Figure B2 shows AUROC for all 15 evaluated models on each of the five ReMedE tasks. Zero-shot generalist and biomedical models underperform on most tasks despite strong mortality prediction. After finetuning, Lang1-1B achieves the highest AUROC on all five tasks.

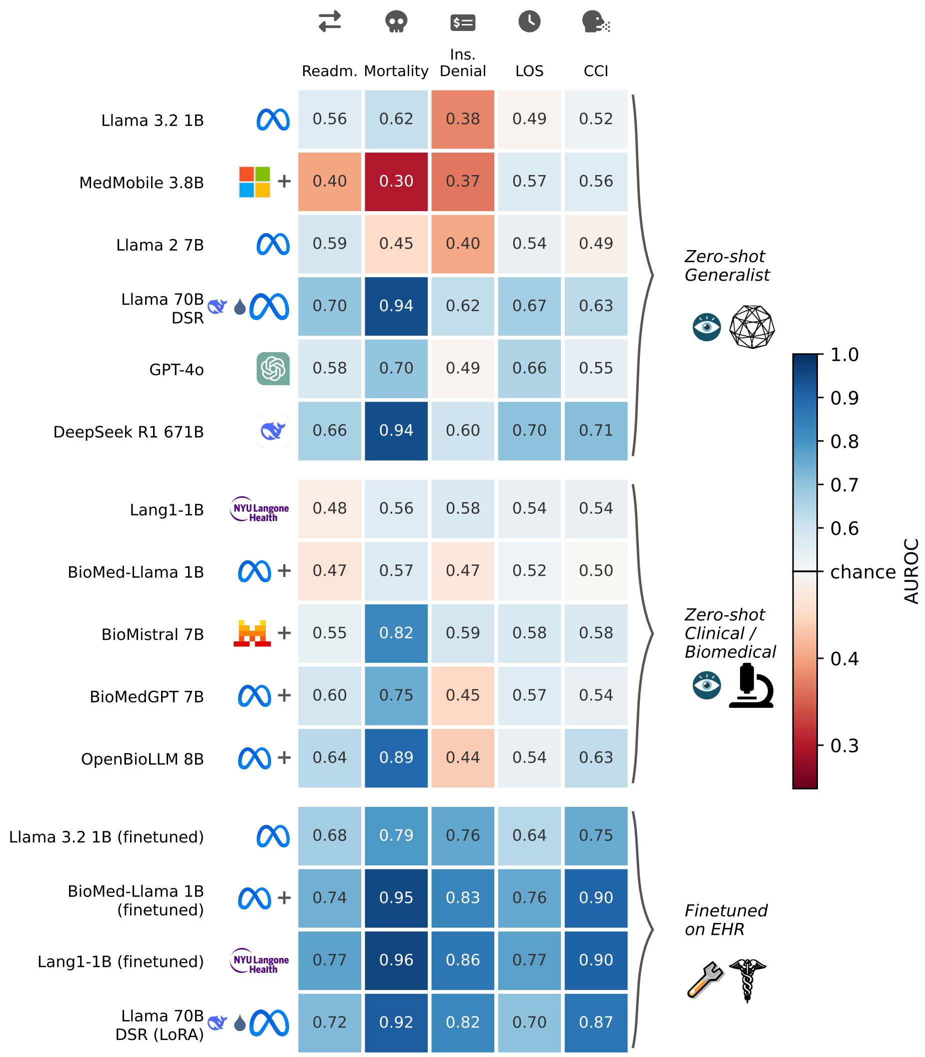

Fig. B2: **Per-task AUROC for all 15 models on ReMedE.** Models are grouped by evaluation setting: zero-shot generalist, zero-shot biomedical, and finetuned. Lang1-1B achieves the highest AUROC on all five tasks after finetuning.

[Figure B3](#) presents per-task performance breakdowns with confidence intervals. Under zero-shot inference ([Figure B3a](#)), both generalists and specialists underperform on most tasks, with mortality as the sole exception. After finetuning ([Figure B3b](#)), LANG1-1B outperforms the best zero-shot performance by 1.66% to 23.66% AUROC and finetuned baselines by 3.64% to 6.75% AUROC.

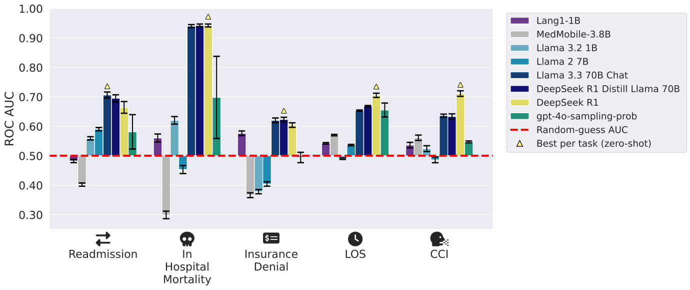

(a) Both generalists and specialists underperform zero-shot. Yellow triangles indicate the best zero-shot performance per task. While the best mortality prediction AUROC is 94.2%, performance on other tasks (readmission, insurance denial, LOS, CCI) ranges from 36.6%–71.7% AUROC.

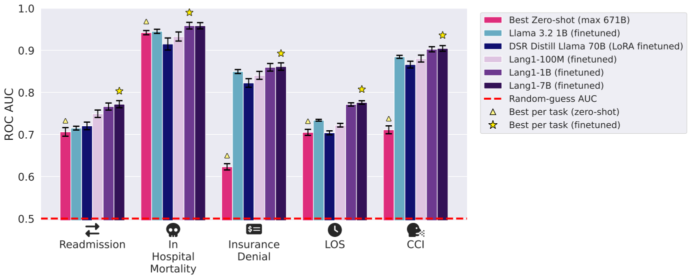

(b) Finetuned LANG1-1B (purple) outperforms best zero-shot performance (magenta) by 1.66% to 23.66% AUROC and finetuned LLAMA 3.2 1B (light blue) and LoRA finetuned LLAMA 70B (deep blue) by 3.64% to 6.75% AUROC. Yellow stars indicate the best finetuned performance per task.

Fig. B3: **Per-task performance on REMEDE with confidence intervals.** (a) Zero-shot evaluation across all models. (b) Finetuned models compared to best zero-shot baselines.

Biomedical pretraining helps at full data but not in the low-data regime. We compare LANG1-1B against BIO-MEDICAL-LLAMA-3.2-1B [50], a domain-adapted variant of LLAMA-3.2-1B further trained on biomedical literature. When finetuned with the full training set, BIO-MEDICAL-LLAMA achieves a mean AUROC of 0.84, nearly matching LANG1-1B (0.85) and outperforming finetuned LLAMA-3.2-1B (0.72). LANG1-1B maintains a consistent per-task advantage across all five tasks. However, the narrow gap at full data obscures a much larger difference in data efficiency: at 100 samples, LANG1-1B leads by 18 points, a gap that narrows to 3 points at full data (Supplementary K). BIO-MEDICAL-LLAMA also shows generally weaker cross-task transfer than LANG1-1B (Supplementary K).

Extended Data C Calibration Plots

Calibration curves are calculated using scikit-learn with $n = 15$ bins. Expected calibration error (ECE) is calculated with $n = 15$ bins using torchmetrics. Single-task calibration curves (Figure C4a) and joint-model calibration curves (Figure C4b) both show close agreement with the ideal diagonal.

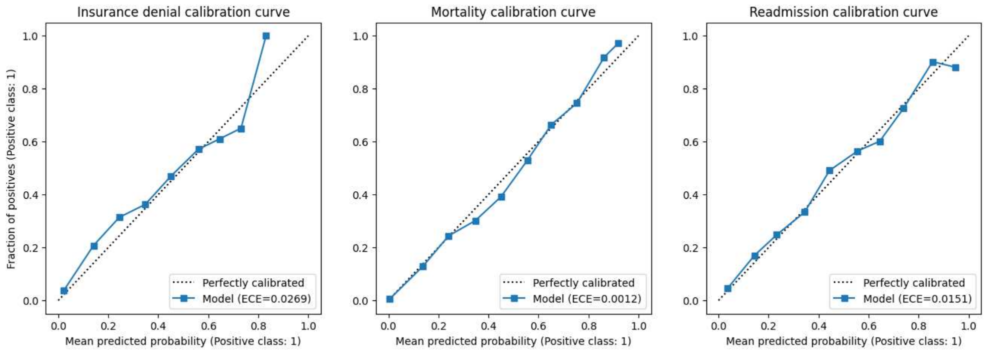

(a) Calibration plots for single-task models.

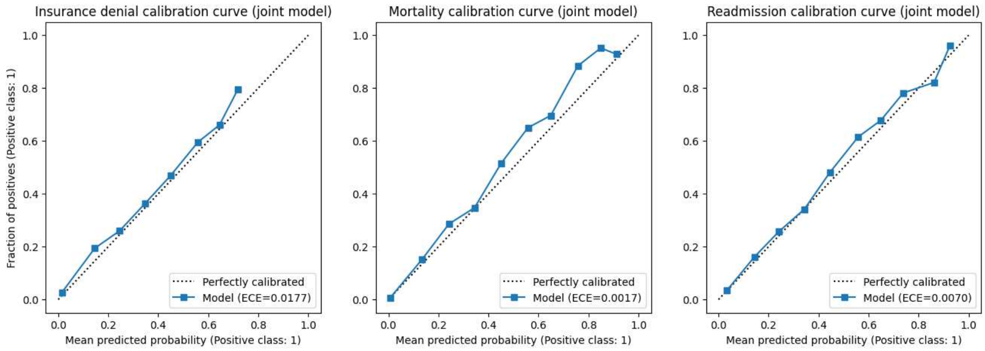

(b) Calibration plots for joint model.

Fig. C4: Both single-task and joint finetuned LANG1 models are well calibrated.

Extended Data D Transfer Pattern of Llama 3.2 1B vs. Lang1 1B

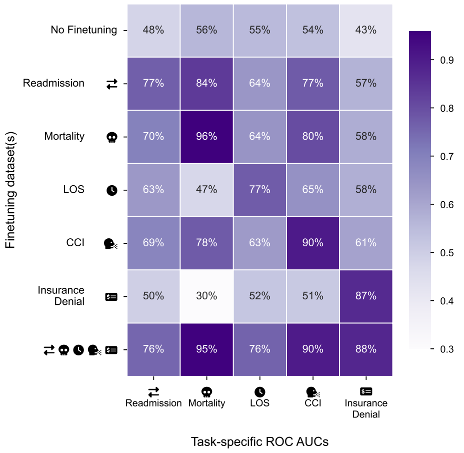

(a) Lang1-1B's transfer heatmap.

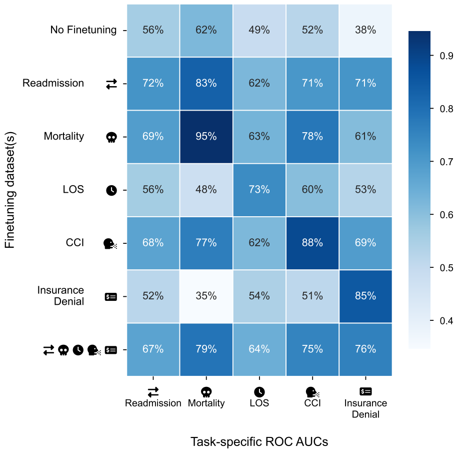

(b) Llama 3.2 1B's transfer heatmap.

Fig. D5: **Lang1-1B and Llama 3.2 1B transfer differently.** The heatmap shows each model's performance when finetuned on a subset of ReMedE tasks (y axis) and evaluated on all five tasks (x axis). Overall, Lang1-1B has higher per-task and joint performance and shows a different transfer pattern than Llama 3.2 1B.

Llama-3.2-1B has overall worse performance than Lang1-1B. Compared to Figure D5a, Figure D5b has worse single-task (diagonal) and joint-task (last row) performance, suggesting that the specific transfer pattern is highly model-specific.

Both models exhibit some similar patterns: (i) finetuning on readmission (second row) boosts performance on the other four tasks, and (ii) transfer can be asymmetric: mortality helps LOS but LOS does not help mortality, which can be explained using domain knowledge (Supplementary M).

Lang1-1B and Llama-3.2-1B also have different patterns: (i) joint finetuning (last row) helps Lang1-1B but hurts Llama-3.2-1B, and (ii) finetuning on insurance denial (fourth row) lowers Lang1-1B's LOS performance while improving it for Llama-3.2-1B. These results suggest that instruction finetuning enables cross-task transfer, though the specific transfer patterns depend on model pretraining. For a comparison of Lang1-1B against Bio-Medical-Llama, see Supplementary K (Figure K16).

Extended Data E External Validation: MIMIC vs. NYU

To check how well LANG1 generalises to a different health system, we compare finetuning LANG1-1B and LLAMA-3.2-1B on three tasks (readmission, mortality, LOS) using data from both MIMIC III and NYU, and evaluate on both health systems. Readmission results are in the main text (Figure 5b); mortality and LOS results are shown in Figure E6.

Finetuning LANG1-1B on NYU transfers to MIMIC-III. The difference in mean AUROC between finetuning on MIMIC III versus NYU ranges from 0.5%–1.8% and is roughly within standard error (Figure 5b, Figure E6).

LANG1-1B achieves better performance than LLAMA-3.2-1B. The AUROC difference ranges from 0.5%–9.6%.

Bidirectional transfer is asymmetric. An alternative view of the same data as transfer matrices (Figure E7) reveals that transferring from MIMIC to NYU and from NYU to MIMIC produce different patterns. For readmission, LANG1-1B finetuned on MIMIC achieves 75.1% on NYU, outperforming the NYU-finetuned model (72.4%), despite MIMIC having fewer labelled pairs. For mortality, LANG1-1B retains 91.6% AUROC on NYU even when finetuned only on MIMIC. For LOS, the in-distribution advantage is more pronounced: NYU-finetuned models perform best on NYU (69.9% vs. 59.3%).

Finetuning on NYU can outperform finetuning on MIMIC for LLAMA-3.2-1B. This is likely because NYU data has more labelled pairs. We confirm this by showing that downsampling NYU to the same size as MIMIC produces similar results for LLAMA-3.2-1B (Figure E6c). This pattern does not hold for LANG1-1B (Figure E6d).

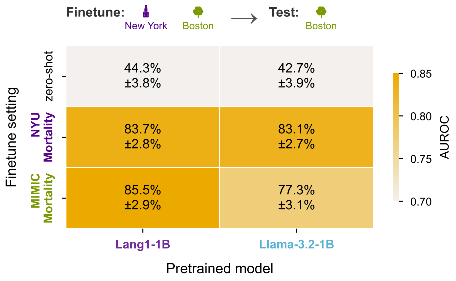

(a) Mortality.

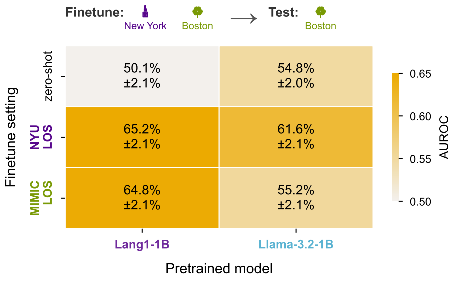

(b) LOS.

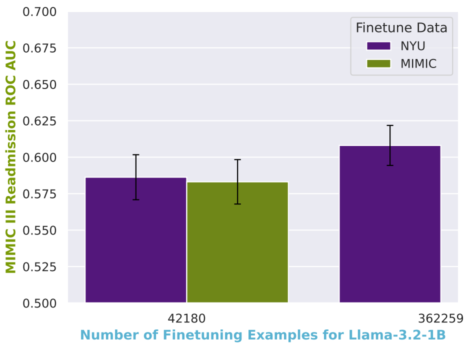

(c) Data efficiency: LLAMA-3.2-1B.

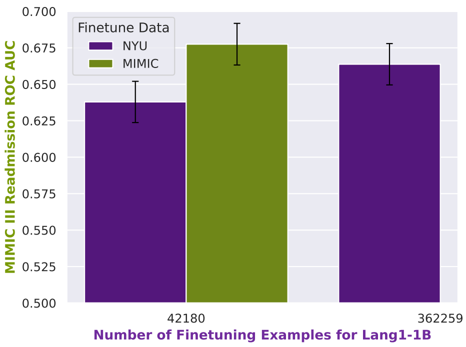

(d) Data efficiency: LANG1-1B.

Fig. E6: **External validation on MIMIC III.** (a,b) Mortality and LOS heatmaps (readmission in Figure 5b). (c,d) Data efficiency ablation: clinical models benefit more from in-domain data.

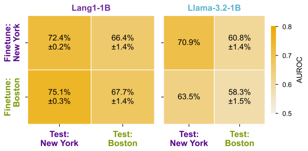

(a) Readmission.

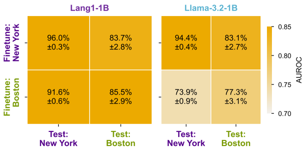

(b) Mortality.

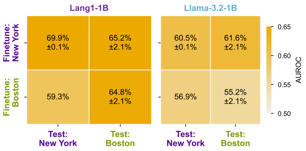

(c) LOS.

Fig. E7: **Cross-system transfer matrices.** LANG1-1B transfers better than LLAMA-3.2-1B in both directions across all three tasks.

Extended Data F Continual Pretraining from Llama

An alternative to pretraining from scratch is *continual pretraining*, where we initialize from an existing general-purpose checkpoint and continue training on clinical data. We compare Lang1-1B pretrained from scratch against a variant initialized from TinyLlama-1.1B (an intermediate checkpoint at step 1,431k, pretrained on 3T tokens of general-domain text) and continually pretrained on the same clinical corpus. Both models are then finetuned on each of the five ReMedE tasks.

Does general pretraining help at step 0? The answer is task-dependent. General pretraining helps for mortality (0.899 vs. 0.854 AUC) and CCI (0.858 vs. 0.847), but does not help for readmission (0.701 vs. 0.728) or LOS (0.589 vs. 0.685). This suggests that representations from general-domain text are more transferable to some clinical tasks than others.

Is continual pretraining more efficient? When treating the 3T tokens of general-domain pretraining as a sunk cost (freely available open-source checkpoint), continual pretraining converges faster on all tasks. However, the efficiency gap narrows with more clinical pretraining. By 50k steps, from-scratch models largely close the gap. If we account for total pretraining tokens, from-scratch Lang1 is the more token-efficient learner, achieving comparable final performance using $\sim$420B tokens vs. 3T+ tokens for continual pretraining.

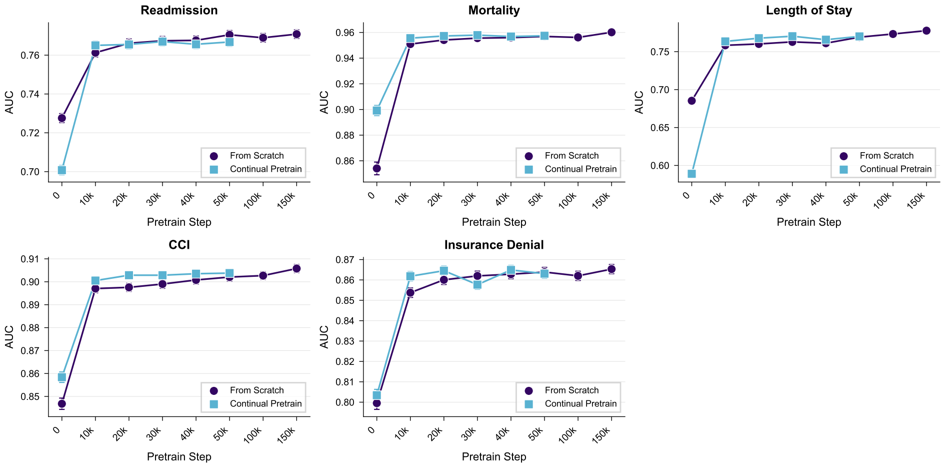

Fig. F8: **Continual pretraining from TinyLlama vs. pretraining from scratch.** Each panel shows finetuned ROC AUC on one ReMedE task as a function of pretraining steps. Continual pretraining (blue) reaches near-peak performance within 10k steps; from-scratch pretraining (purple) requires 50k+ steps to converge. Error bars indicate 95% bootstrap confidence intervals.

Extended Data G Stratified Evaluation

We performed stratified evaluation on readmission to evaluate performance variation across demographic groups (age, first race, borough, ethnicity, sex, and whether the patients are children). Some groups are omitted due to single-class representation from small sample size.

To test whether model performance varies across subgroups, we use a two-level procedure with Bonferroni correction at each level. First, we run a one-way ANOVA F-test within each of the six demographic categories, with the significance threshold adjusted to $\alpha = 0.05/6 \approx 0.0083$ to control the family-wise error rate across categories. Four categories show significant variation: age ($F(16,\ 37{,}009) = 8.34$, $p < 0.001$), borough ($F(5,\ 35{,}862) = 6.52$, $p < 0.001$), sex ($F(1,\ 35{,}928) = 13.64$, $p < 0.001$), and pediatric status ($F(1,\ 37{,}935) = 27.66$, $p < 0.001$); first race ($F(8,\ 35{,}496) = 1.59$, $p = 0.12$) and ethnicity ($F(3,\ 7{,}940) = 1.80$, $p = 0.15$) are not significant. For categories where the omnibus ANOVA is significant, we then perform post-hoc two-sided z-tests comparing each subgroup's AUC to the sample-size-weighted grand mean, with p-values multiplied by the number of subgroups in that category (Bonferroni correction). Without this correction, the probability of at least one spurious significant result would increase with the number of tests, potentially leading to false claims of demographic disparity.

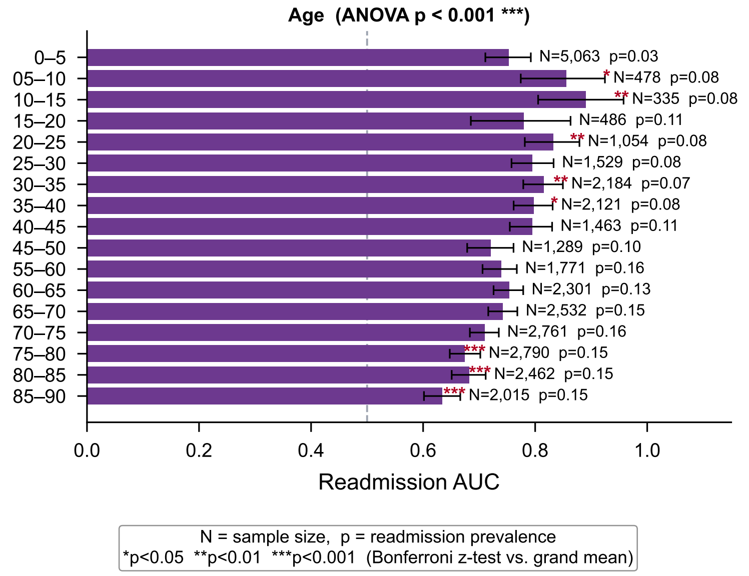

Fig. G9: **Stratified readmission AUC by age.** Bars show temporal test AUC per subgroup with bootstrap 95% CIs. Red stars mark subgroups significantly different from the weighted grand mean (z-test, Bonferroni-corrected); ANOVA p-value in panel title ($p < 0.001$). N: sample size; p: readmission prevalence.

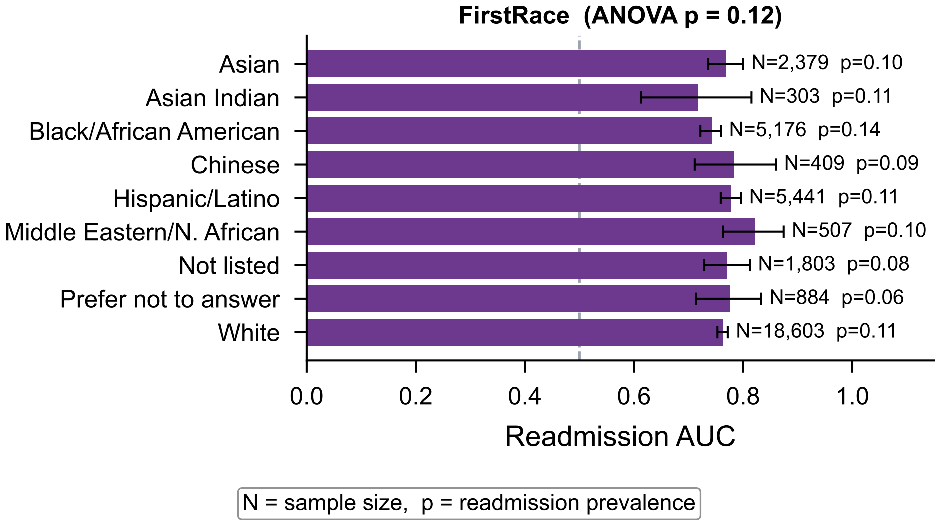

(a) Race.

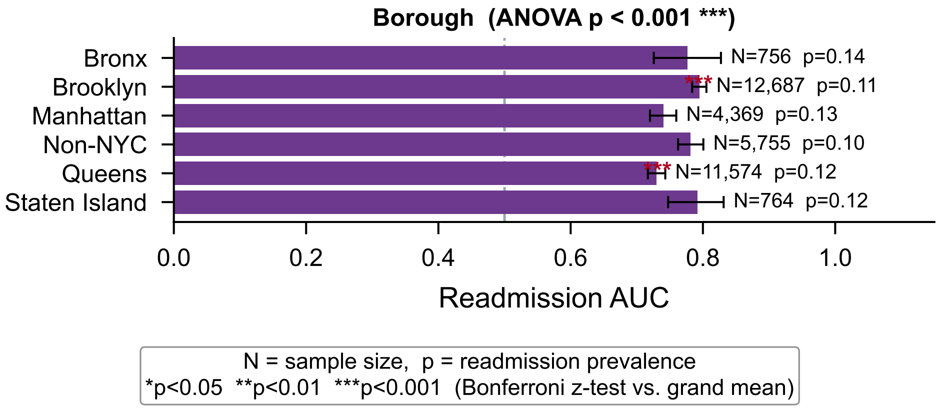

(b) Borough.

Fig. G10: **Stratified readmission AUC by race and borough.** Bars show temporal test AUC per subgroup with bootstrap 95% CIs. Red stars mark subgroups significantly different from the weighted grand mean (z-test, Bonferroni-corrected); ANOVA p-value in each panel title (both $p < 0.001$). N: sample size; p: readmission prevalence.

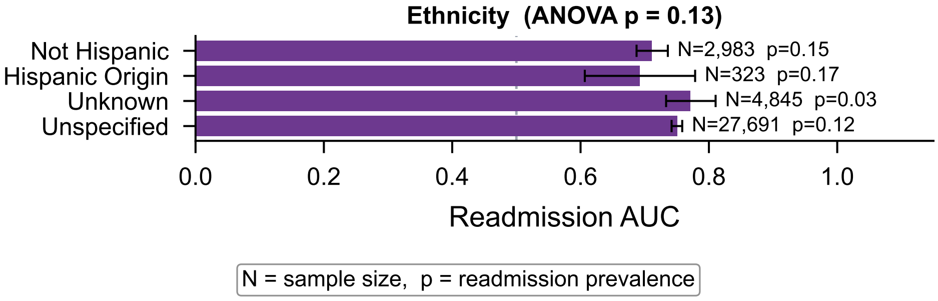

(a) Ethnicity.

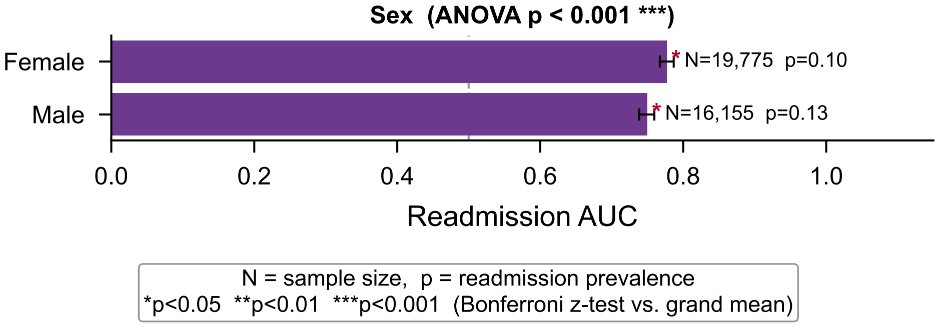

(b) Sex.

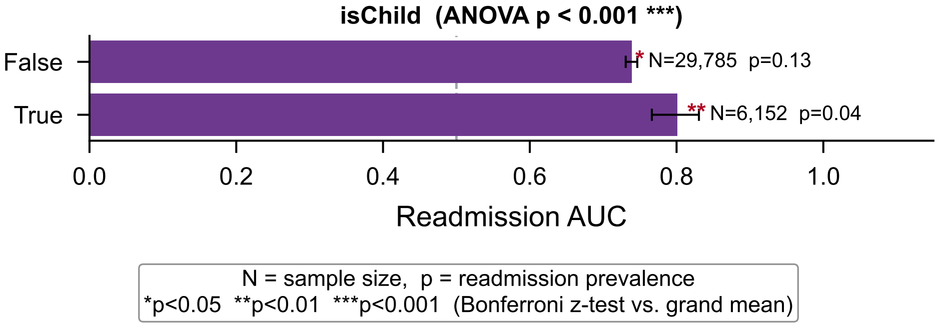

(c) Pediatric status.

Fig. G11: **Stratified readmission AUC by ethnicity, sex, and pediatric status.** Bars show temporal test AUC per subgroup with bootstrap 95% CIs. Red stars mark subgroups significantly different from the weighted grand mean (z-test, Bonferroni-corrected); ANOVA p-value in each panel title. AUC varies significantly across sex and pediatric status ($p < 0.001$), but not ethnicity ($p = 0.13$). N: sample size; p: readmission prevalence.

Extended Data H Effect of Context Length on Zero-Shot Performance

All main-text results truncate inputs to 512 tokens for fair comparison with NYUTRON [23]. To assess whether this truncation biases our findings, we repeat the zero-shot evaluation at each model's full native sequence length across all five REMEDE tasks and all seven baseline models.

The extent of truncation is substantial (Figure H12a): the median readmission discharge note contains 1,700 tokens in the training set and 2,573 tokens in the new temporal test set (Llama-2 tokenizer), meaning 512-token truncation discards 70–80% of the median note. Moreover, 88% of training notes and 99% of temporal test notes exceed 512 tokens, so truncation affects nearly every example. The shift toward longer notes in the temporal test set likely reflects evolving documentation practices (e.g., template expansion) and means that the 512-token window captures a shrinking fraction of newer clinical records.

Despite this, Figure H12b shows that paired AUROC values at 512 tokens versus full sequence are largely stable across model–task combinations. A Wilcoxon signed-rank test over all 35 pairs finds no significant difference ($W = 255.5$, $p = 0.33$; median ΔAUC $= -0.006$, mean ΔAUC $= -0.006$). Per-task tests are likewise nonsignificant (all $p > 0.10$). Spearman rank correlation between the two conditions is $\rho = 0.91$ ($p < 10^{-6}$), confirming that the relative model ranking is preserved. Per-task correlations range from $\rho = 0.82$ (Insurance Denial) to $\rho = 1.00$ (CCI), all significant at $p < 0.025$.

Individual model–task differences are generally small ($|\Delta| < 0.03$), with a few exceptions: LLAMA-2-7B gains $+0.148$ on mortality when given the full note (rising from near-chance 0.488 to 0.636, still well below frontier models at ~0.93), while LLAMA-3.3-70B-CHAT drops -0.099 on insurance denial. In several cases, full-sequence performance is *worse* than truncated performance. One possible explanation is that clinical notes tend to front-load the most relevant information, while later sections contain lower-signal content; longer contexts may also introduce distractor dilution. Regardless of the mechanism, these results indicate that our findings are not sensitive to the 512-token truncation choice: even though only 20–30% of the median note is retained, model rankings and performance differences are preserved. We conclude that the 512-token truncation is a reasonable evaluation design choice that does not systematically favor or penalize any model class.

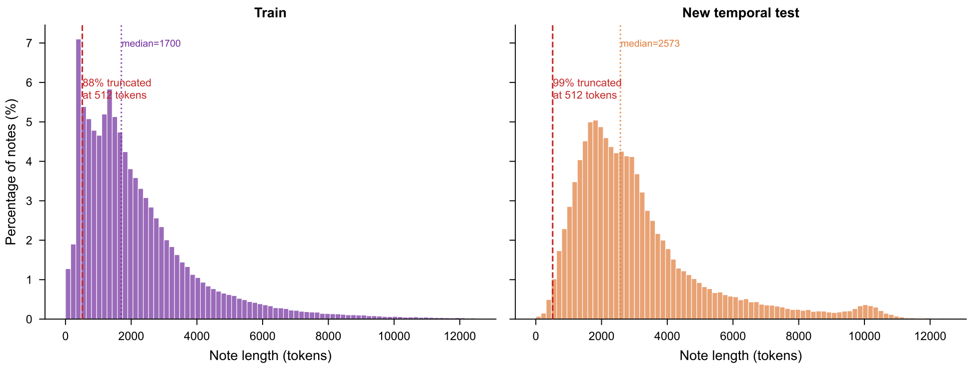

(a) Token-length distribution of readmission discharge notes. Dashed red lines mark the 512-token threshold; dotted lines indicate the median. Temporal test notes are substantially longer (median 2,573 vs. 1,700 tokens).

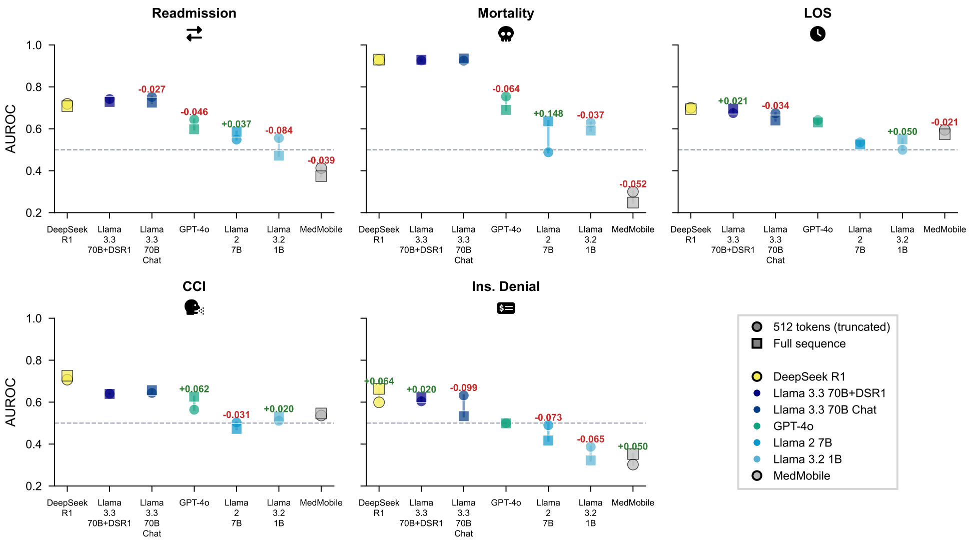

(b) Zero-shot AUROC at 512 tokens vs. full sequence length. The model ranking is highly preserved (Spearman $\rho = 0.91$, $p < 10^{-6}$).

Fig. H12: **512-token truncation does not bias conclusions.** (a) Note length distributions. (b) Paired AUROC comparison: circles denote 512-token truncation; squares denote full native sequence. Green annotations indicate improvement; red indicates degradation. Only differences > 0.02 are annotated.

Supplementary I Pretraining vs. Finetuning Trajectory for All Tasks

Figure 4a in the main text demonstrates the pretraining–finetuning token efficiency trade-off for readmission. Here we present the same analysis for the remaining four ReMedE tasks.

Mortality (Figure I13a). Shows the largest pretraining benefit (∼11 AUC points between no-pretraining and full-pretraining configurations).

Length of Stay (Figure I13b). Pretraining contributes ∼5 AUC points above finetuning alone. The lowest finetuning budget performs near random chance.

CCI (Figure I13c). Has the highest baseline (∼0.85 AUC without pretraining). Full pretraining adds ∼4 points.

Insurance Denial (Figure I13d). Shows a moderate pretraining benefit of ∼5 AUC points. The lowest finetuning budget stays below chance, reflecting the institution-specific nature of this task.

Across all four tasks, two consistent findings hold: (1) at any fixed total token budget, more finetuning tokens yield higher downstream AUC; and (2) pretraining still provides value beyond what finetuning alone achieves, with a gap of 4–11 AUC points depending on the task.

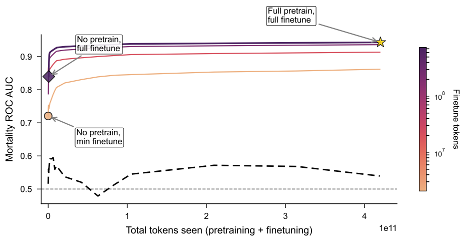

(a) Mortality (∼11 AUC point pretraining benefit).

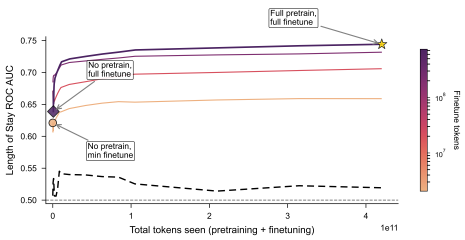

(b) Length of stay (∼5 AUC point benefit).

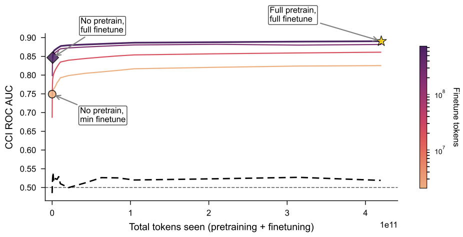

(c) CCI (∼4 AUC point benefit).

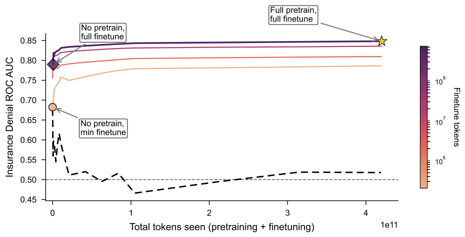

(d) Insurance denial (∼5 AUC point benefit).

Fig. I13: **Pretraining vs. finetuning trajectory for all remaining ReMedE tasks.** Each panel shows finetuned AUC as a function of total tokens (pretraining + finetuning). Pretraining provides 4–11 AUC points above finetuning alone across all tasks; at any fixed budget, more finetuning tokens yield higher AUC.

Supplementary J Instruction Finetuning Ablation

One possible explanation for near-random zero-shot clinical performance is that the pretrained model simply does not understand the task prompt. To test this hypothesis, we finetuned LANG1-1B checkpoints on the OpenAssistant Conversations v2 dataset (OASST2) and re-evaluated zero-shot clinical performance. OASST2 contains 12,947 English conversation trees, providing broad instruction-following supervision. We finetuned each pretrained checkpoint for 200 steps using a learning rate of 3.264×10^{-4}, an effective batch size of 1024, AdamW with β_1=0.85, β_2=0.98, weight decay of 0.01, and a warmup ratio of 0.1. During finetuning, we masked the prompt tokens so that the loss is computed only on the assistant response.

Figure J14 compares zero-shot clinical performance across pretraining for instruction-finetuned vs. base (non-IFT) checkpoints on five clinical classification tasks and three reading comprehension benchmarks. After instruction finetuning, clinical AUROC remains close to random chance, closely tracking the non-IFT trajectory throughout pretraining. This confirms that the failure to classify clinical outcomes zero-shot is not due to a lack of instruction understanding but reflects a fundamental gap: the mapping from clinical notes to operational labels is not present in the pretrained representations and must be learned through task-specific supervision.

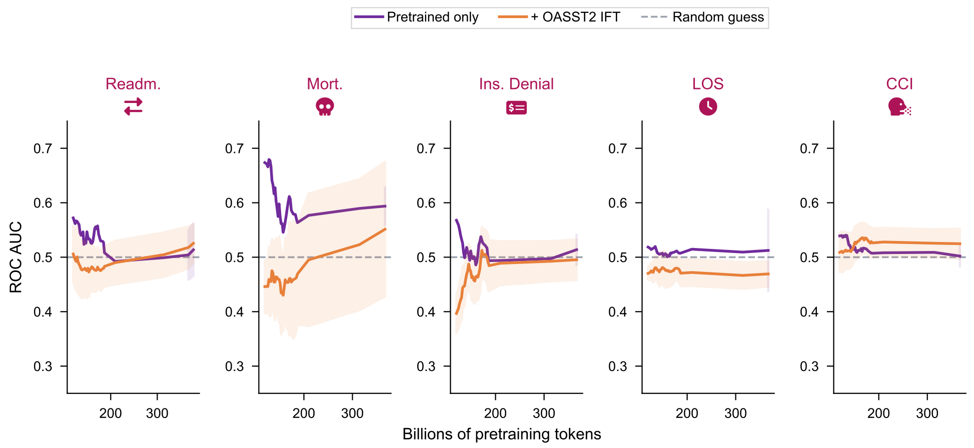

Fig. J14: **Instruction finetuning does not improve zero-shot clinical classification.** Comparison of zero-shot eval-harness performance across pretraining for LANG1-1B with (red) and without (blue) OASST2 instruction finetuning. Each dot is a single checkpoint evaluation; lines show EWM-smoothed trends. Clinical AUROC (top row) remains near chance regardless of instruction finetuning.

Supplementary K Bio-Medical-Llama Data Efficiency and Transfer

Lang1-1B outperforms Bio-Medical-Llama-3.2-1B [50] at every sample size from 100 to 362,259 (Figure K15), with the largest gains in the low-data regime, indicating that adaptation on published biomedical text does not substitute for pretraining on clinical notes.

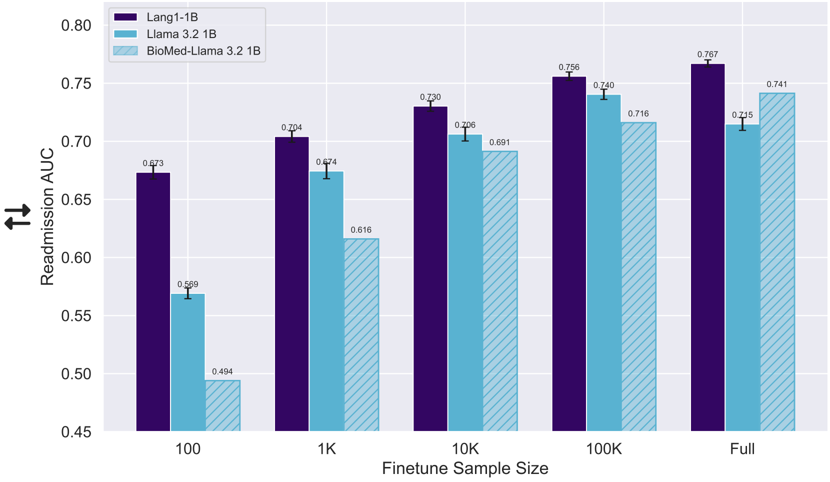

Fig. K15: **Biomedical domain adaptation does not substitute for clinical pretraining.** Readmission AUROC on the 2024 temporal test set for Lang1-1B, Llama-3.2-1B, and Bio-Medical-Llama-3.2-1B across five finetune sample sizes and the full training set (n=362,259). Lang1-1B outperforms both baselines at every sample size. Bio-Medical-Llama surpasses Llama-3.2-1B only at full data.

Bio-Medical-Llama shows generally weaker cross-task transfer than Lang1-1B. Figure K16 compares the transfer heatmaps for Lang1-1B and Bio-Medical-Llama-3.2-1B side by side. While Bio-Medical-Llama achieves competitive single-task (diagonal) performance (mean diagonal 83.5% vs. 85.2%, a gap of 1.6 pp), the off-diagonal gap is larger: Lang1-1B has higher off-diagonal transfer in 16 of 20 cells, with a mean off-diagonal AUROC of 63.6% vs. 61.1% (+2.5 pp). The advantage is largest when finetuning on mortality (mean off-diagonal 70.8% vs. 64.8%, +6.0 pp) and readmission (72.8% vs. 69.2%, +3.7 pp). The four cells where Bio-Medical-Llama has higher off-diagonal transfer are all small ($\leq$1.9 pp): three are in the insurance denial finetuning row, where both models are near chance, and one is CCI$\rightarrow$insurance denial (0.1 pp). These results suggest that biomedical literature pretraining improves per-task performance but does not confer the same cross-task transfer benefits as clinical note pretraining. See also Extended Data D for the analogous Llama 3.2 1B comparison.

Joint finetuning reveals a gradient of multi-task robustness across pretraining strategies. The last row of each transfer heatmap shows the model jointly finetuned on all five tasks. Lang1-1B retains near-parity with its single-task diagonal (mean joint AUROC 85.0% vs.

85.4%, a drop of 0.4 pp), whereas Bio-Medical-Llama drops from 83.6% to 80.2% (−3.4 pp) and Llama-3.2-1B drops from 82.6% to 72.2% (−10.4 pp). The degradation in Llama-3.2-1B is especially pronounced for mortality (95%→79%, −16 pp) and CCI (88%→75%, −13 pp). Bio-Medical-Llama falls between the two: its biomedical pretraining partially buffers against this degradation (largest per-task drop 5 pp on LOS), but does not match the stability of clinical note pretraining. These results suggest that domain-specific pretraining on clinical notes better enables multi-task learning than general-purpose or biomedical-literature pretraining.

Comparison with NYUTron. NYUTron is not included in the ReMedE comparison plots because ReMedE is designed for evaluating generative (decoder-only) models, whereas NYUTron is an encoder-based model with a different inference pipeline. On the same 2024 temporal test set, Lang1-1B achieves a slightly higher mean AUROC than NYUTron across all five tasks (85.2% vs. 84.9%, +0.35%), with per-task AUROCs of 76.7% vs. 74.1% (readmission), 95.9% vs. 95.8% (mortality), 86.0% vs. 86.4% (insurance denial), 77.1% vs. 77.7% (LOS), and 90.3% vs. 90.3% (CCI). Lang1 is higher on readmission and mortality, comparable on CCI, and slightly lower on insurance denial (−0.4 pp) and LOS (−0.6 pp). Critically, Lang1 achieves this from a single jointly finetuned model that additionally supports cross-task transfer and cross-site generalisation, whereas NYUTron requires a separately trained model per task.

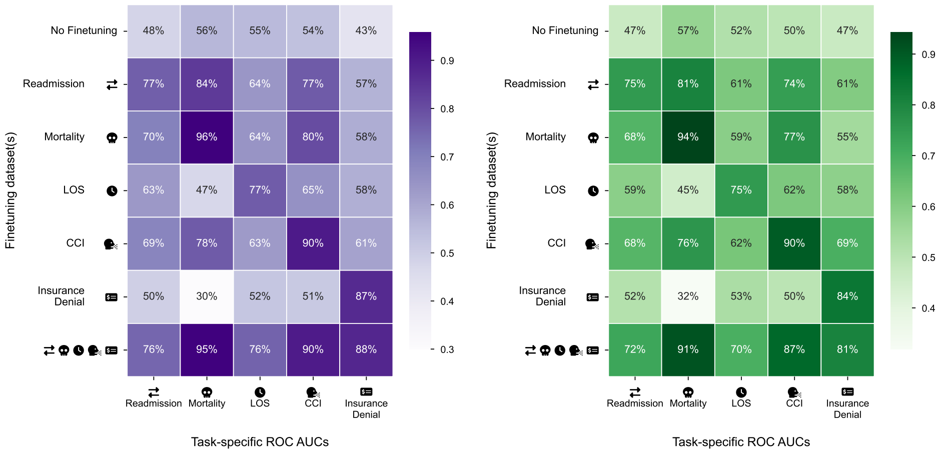

(a) Lang1-1B's transfer heatmap. (b) Bio-Medical-Llama 1B's transfer heatmap.

Fig. K16: **Lang1-1B vs. Bio-Medical-Llama 1B cross-task transfer.** Each row represents the finetuning task(s); each column shows evaluation AUROC on all five ReMedE tasks. Bio-Medical-Llama achieves competitive diagonal performance but generally weaker off-diagonal transfer than Lang1-1B (16 of 20 off-diagonal cells), suggesting that biomedical literature pretraining is less effective than clinical note pretraining for enabling cross-task generalisation.

Supplementary L   Pretraining Ablations

Figure L17 presents pretraining ablation results evaluated on the 2024 readmission temporal test set. We control for model architecture (encoder vs. decoder), model size (100M vs. 1B), and pretraining data (NYU Notes, NYU Notes+, NYU Notes+ and web texts). We evaluate on three clinical predictive tasks chosen for their distinct transfer patterns.

Training larger models on more recent clinical data improves temporal robustness. Figure L17 shows readmission ablation results. Compared to models trained only on EHR from 2013 to 2021 ($\mathcal{D}_{\text{NYUNotes}}$), adding more recent clinical data ($\mathcal{D}_{\text{NYUNotes+}}$) and further mixing in general-domain text ($\mathcal{D}_{\text{NYUNotes+,WebText}}$) improves performance for the 1B model but not the 100M model, suggesting that larger models are better able to use additional clinical data. This also justifies our choice of equally mixing EHR and web texts, since adding web texts does not significantly hurt clinical task performance while instilling general-purpose knowledge. Similar patterns are observed for insurance denial (Figure L18a) and LOS (Figure L18b).

Scaling from 1B to 7B yields diminishing returns. After finetuning, Lang1-7B achieves mean AUROC of 85.4% (readmission 77.2%, mortality 95.8%, insurance denial 86.1%, LOS 77.6%, CCI 90.5%), compared with 85.2% for Lang1-1B. The per-task improvements are $\leq$0.5 percentage points, indicating that at this pretraining data scale, model capacity is not the primary bottleneck for clinical classification performance.

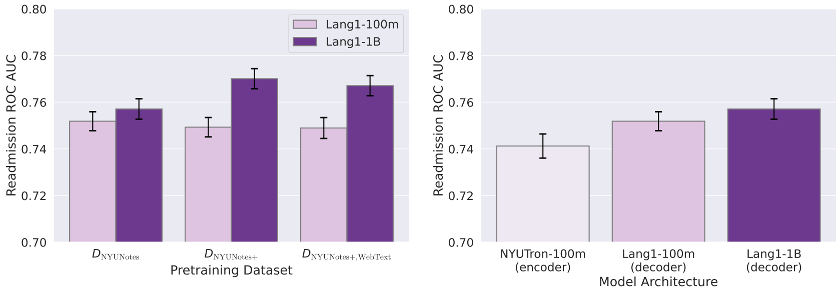

Fig. L17: **Pretraining ablations: readmission prediction.** Error bars indicate 95% confidence intervals. Larger models trained on more clinical data perform better, and mixing web texts does not hurt substantially.

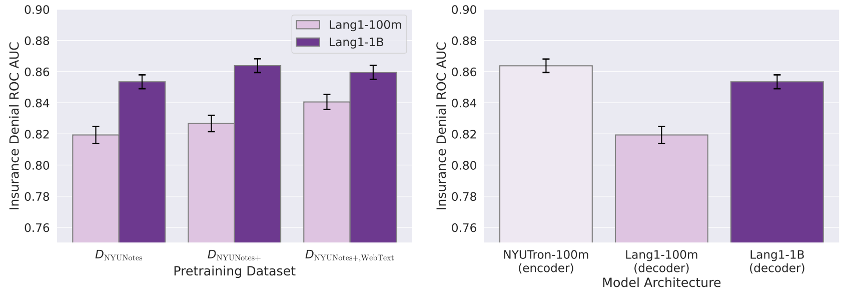

(a) Insurance denial prediction.

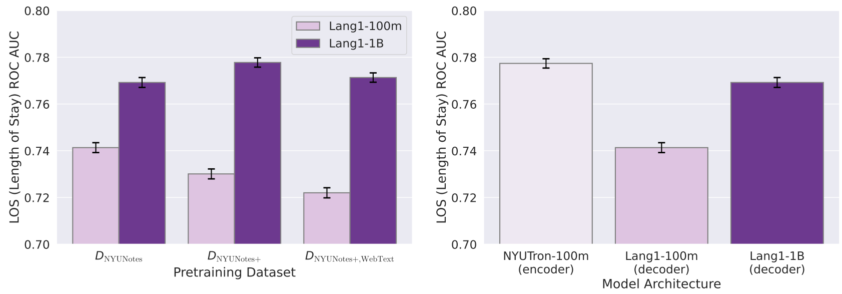

(b) LOS prediction.

Fig. L18: **Pretraining ablations: insurance denial and LOS.** Similar patterns are observed as for readmission.

Supplementary M Asymmetry of Transfer between Mortality and LOS

Our medical collaborators provided an explanation for the asymmetric transfer between mortality and LOS observed for both LANG1-1B and LLAMA-3.2-1B. Figure M19 shows the conditional probability analysis. If a patient died, they either stayed for a short time (very sick, died immediately) or a long time (doctors failed to save them; Figure M19a). On the other hand, if a patient stayed for a long time, they either survived or died after doctors' attempts (Figure M19b). This asymmetry in conditional probability helps explain why mortality transfers to LOS but not vice versa.

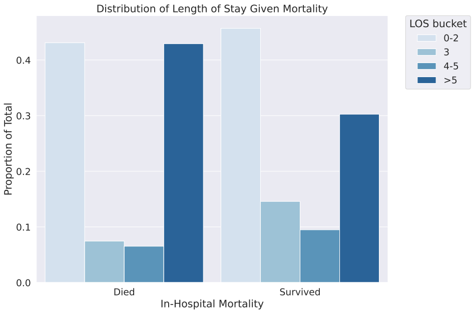

(a) Probabilities of LOS given in-hospital mortality.

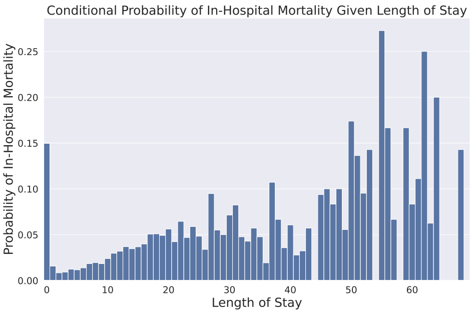

(b) Probabilities of in-hospital mortality given LOS.

Fig. M19: **Conditional probability analysis explains the asymmetry of transfer** between mortality and LOS.

Supplementary N ReMedE Dataset Statistics

The following tables show the note counts (Table N1) and patient counts (Table N2) across each split, and class ratios for each task: readmission (Table N3), mortality (Table N4), length of stay (Table N5), comorbidity imputation (Table N6), and insurance denial (Table N7).

Table N1: Distinct note counts for each ReMedE task across five splits.

Task	Train	Val	Test	Temporal Test 2021	Temporal Test 2024	Total
Readmission	362,259	45,282	45,283	53,916	97,586	604,326
CCI	256,676	32,085	32,085	42,137	80,932	443,915
Length of Stay	334,515	41,814	41,815	51,018	97,586	566,748
Insurance Denial	41,842	2,325	2,325	9,299	42,046	97,837
Mortality	334,515	41,814	41,815	51,018	97,586	566,748

Table N2: Distinct patient counts for each ReMedE task across five splits.

Task	Train	Val	Test	Temporal Test 2021	Temporal Test 2024	Total
Readmission	269,140	42,692	42,603	46,003	78,453	421,429
CCI	188,298	30,085	30,098	251,804	64,873	306,741
Length of Stay	248,486	39,304	39,331	43,358	78,453	395,991
Insurance Denial	39,422	2,319	2,313	9,037	37,821	87,974
Mortality	248,674	39,317	39,357	43,358	78,453	395,991

Table N3: Readmission label ratios by split (Total notes = 604,326; total words = 607,877,177).

Split	Not Readmitted	Readmitted within 30 days
Train	0.891495	0.108505
Val	0.891944	0.108056
Test	0.893293	0.106707
Temporal Test (2021)	0.888456	0.111544
New Temporal (2024)	0.887115	0.112885

Table N4: In-hospital mortality label ratios by split (Total notes = 566,748; total words = 608,603,182).

Split	Survived	Died
Train	0.981161	0.018839
Val	0.981250	0.018750
Test	0.980916	0.019084
Temporal Test (2021)	0.980693	0.019307
New Temporal (2024)	0.982241	0.017759

Table N5: Length of stay label ratios by split (Total notes = 566,748; total words = 608,603,182).

Split	0–2 days	3 days	4–5 days	>5 days
Train	0.417306	0.176575	0.165888	0.240231
Val	0.415722	0.180562	0.164490	0.239226
Test	0.414851	0.176611	0.167452	0.241086
Temporal Test (2021)	0.418597	0.153201	0.164805	0.263397
New Temporal (2024)	0.456418	0.144529	0.159757	0.239297

Table N6: Charlson Comorbidity Index (CCI) label ratios by split (Total notes = 443,915; total words = 524,739,038).

Split	Score 0	Score 1–2	Score 3–4	Score 5–7	Score >7
Train	0.694681	0.224840	0.054251	0.025324	0.000904
Val	0.689169	0.229547	0.055166	0.025526	0.000592
Test	0.693502	0.226866	0.053327	0.025370	0.000935
Temporal Test (2021)	0.685811	0.228327	0.059164	0.025821	0.000878
New Temporal (2024)	0.684043	0.217355	0.067513	0.029370	0.001717

Table N7: Insurance denial label ratios by split (Total notes = 97,837; total words = 89,147,715).

Split	Approved (0)	Denied (1)
Train	0.877850	0.122150
Val	0.867097	0.132903
Test	0.873978	0.126022
Temporal Test (2021)	0.879880	0.120120
New Temporal (2024)	0.861033	0.138967

Supplementary O Control for Patient Overlap

We chose to construct the 2024 temporal test set without explicit patient split, because patients do come back to the health system at deployment. 14.1% of readmission test encounters, 15.5% of mortality test encounters, and 15.5% of LOS test encounters involve patients whose earlier encounters appeared in the finetuning training or validation sets. We performed an ablation excluding these seen patients: keeping the finetuned model fixed and varying the test data to include or exclude seen patients, we find similar test performance on readmission, mortality, and LOS prediction.

Figure O20a shows that on readmission, removing patients seen from pretraining and finetuning slightly increases performance from 76.5% to 77% AUROC. The increase is 0.39% for mortality (Figure O20b) and 0.70% for LOS (Figure O20c). The slight increase could be attributed to repeated patients being both older (13–16 years older on average) and more likely to be a minority (41% vs. 36% non-white). These findings confirm that our temporal split does not overestimate model performance.

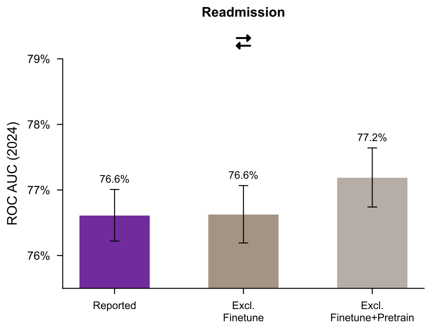

(a) Readmission.

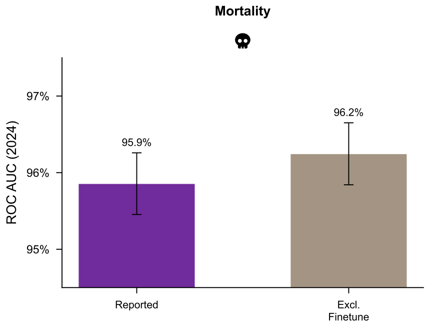

(b) Mortality.

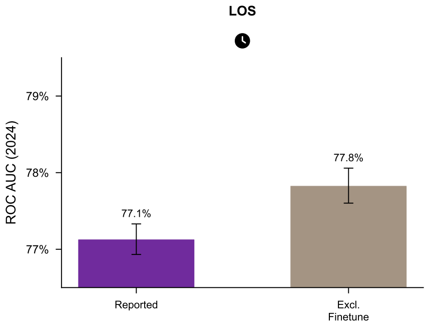

(c) LOS.

Fig. O20: **LANG1-1B's finetuned performance with or without patient split** on the 2024 temporal test set for readmission, mortality, and LOS.

Supplementary P Prompts of ReMedE Tasks

We constructed prompts to create task-specific questions and answer options from the labelled finetuning notes:

- **Readmission** Question: Given the above discharge note of the patient, will the patient be readmitted to the hospital within 30 days of discharge? \n A. no \n B. yes \n Answer:
- **In-Hospital Mortality** Question: Given the above admission note of the patient, will the patient die during the hospital admission? \n A. no \n B. yes \n Answer:
- **Charlson Comorbidity Index** Question: Given the above admission note of the patient, what's the Charlson Comorbidity Index of the patient? \n A. score 0 \n B. score 1 to 2 \n C. score 3 to 4 \n D. score 5 to 7 \n E. score more than 7 \n Answer:
- **Insurance Denial** Question: Given the above discharge note of the patient, will the insurance claim of the patient be denied? \n A. no \n B. yes \n Answer:
- **Length of Stay** Question: Given the above admission note of the patient, how long will the patient stay at the hospital? \n A. 0 to 2 days \n B. 3 days \n C. 4 to 5 days \n D. more than 5 days \n Answer:

Supplementary Q Detailed Statistics of NYU Notes+

We analysed the top five clinical departments, diagnoses, and boroughs for pathology notes, radiology notes, and hospital notes. See Figs. Q21–Q23.

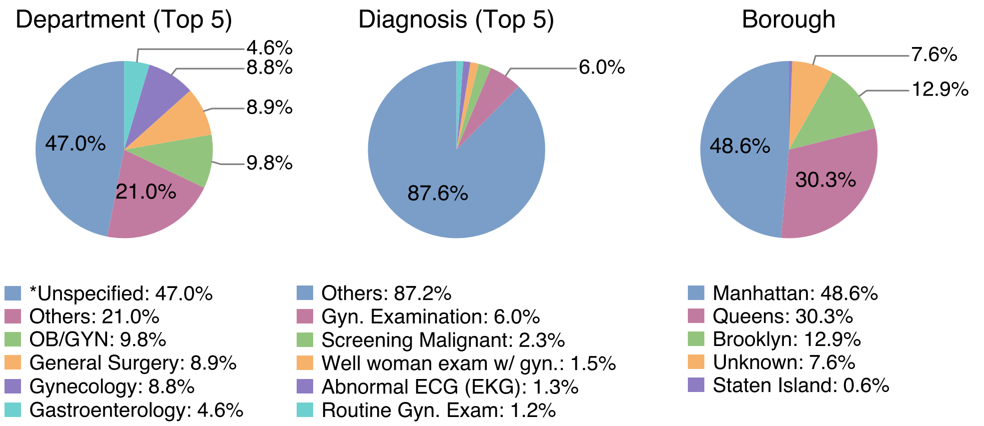

Fig. Q21: **Top five departments, diagnoses, and boroughs of pathology notes in NYU Notes+.** Among pathology notes with specified departments, OB/GYN has the highest percentage (9.8%). The most common specified diagnosis is gynaecological exams (6%). Nearly half (48.6%) of the notes are from Manhattan.

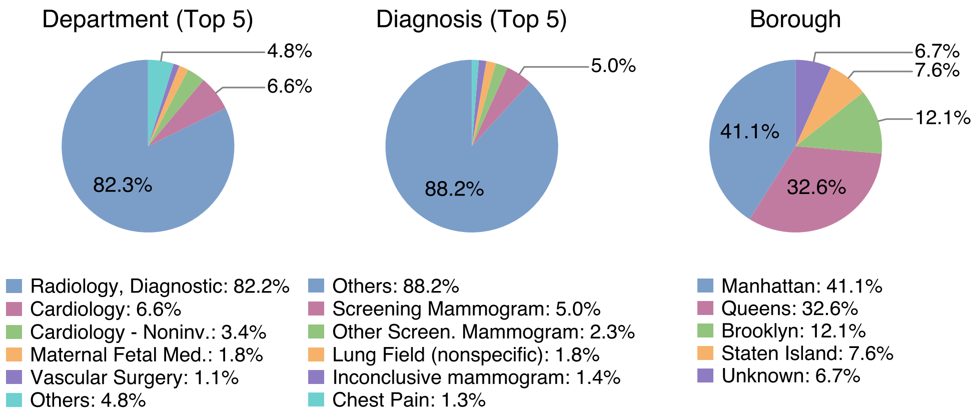

Fig. Q22: **Top five departments, diagnoses, and boroughs of radiology notes in NYU Notes+.** Most radiology notes are from diagnostic radiology (82.2%), with screening mammograms as the most common specified diagnosis (5%). The two most common boroughs are Manhattan (41%) and Queens (32.6%).

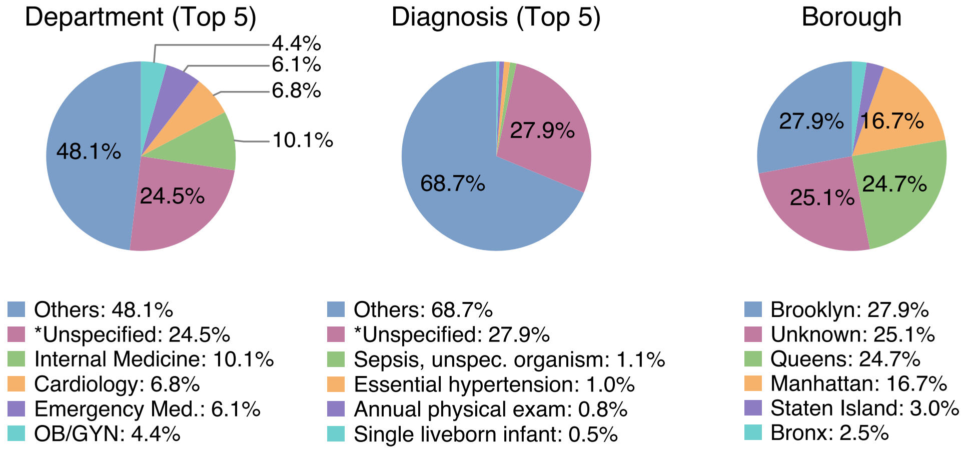

Fig. Q23: **Top five departments, diagnoses, and boroughs of hospital notes in NYU Notes+.** Among notes with specified departments, most are from internal medicine (10.1%), with common diagnoses including sepsis (1.1%) and hypertension (1%). The majority of notes are from Brooklyn (27.9%) and Queens (24.7%).

Supplementary R LoRA Finetuning for Llama-3-70B

To efficiently train DeepSeek-R1-Distill-Llama-3-70B on 1 node of 8 H100s, we used Low-Rank Adaptation (LoRA) finetuning. LoRA reduces trainable parameters by inserting trainable rank decomposition matrices into transformer layers while freezing the pretrained weights. We enabled LoRA adapters on the query and value projections with rank $r = 8$, scaling factor $\alpha = 16$, and dropout rate of 0.05.

Supplementary S Token Probability Approximation for Models without Logprobs

GPT-4o does not provide logprobs. To approximate its probabilities, we sample 10 generations at temperature 1 and count the occurrences of each multiple choice option, normalizing the counts to probabilities. For cost reasons, we limit evaluation to 1,000 examples except for CCI, where we use 10,000 greedy evaluations due to the skewed label distribution.
